# Multiple Attack to Inflorescences of an Annual Plant Does Not Interfere with the Attraction of Parasitoids and Pollinators

**DOI:** 10.1007/s10886-020-01239-6

**Published:** 2021-01-28

**Authors:** Lucille T. S. Chrétien, Hessel van der Heide, Liana O. Greenberg, David Giron, Marcel Dicke, Dani Lucas-Barbosa

**Affiliations:** 1 Laboratory of Entomology, Wageningen University, Droevendaalsesteeg 1, Radix building, Wageningen, 6708PB The Netherlands; 2 UMR 7261, Institut de Recherche sur la Biologie de l’Insecte (IRBI), CNRS/Université François-Rabelais de Tours, Avenue Monge, Parc Grandmont, 37200 Tours, France; 3 Biology Department, École Normale Supérieure de Lyon (ENS L), 46 Allée d’Italie, 69007 Lyon, France; 4 Present Address: School of Biological and Marine Sciences, University of Plymouth, Drake Circus, Plymouth, PL4 8AA UK; 5 Environmental Studies Department, New College of Florida, 5800 Bay Shore Road, Sarasota, FL 34243 USA; 6 Present Address: Bio-communication & Ecology, ETH Zürich, Schmelzbergstrasse 9, 8092 Zürich, Switzerland

**Keywords:** *Brassica nigra* (Brassicaceae), Flowering plants, Indirect resistance, Multiple attack, Plant volatiles, Pollination

## Abstract

Plants in the flowering stage need to ensure reproduction by protecting themselves from attack and by preserving interactions with mutualist pollinators. When different plant mutualists are using the same type of cues, such as volatile compounds, attraction of parasitoids and pollinators may trade off. To explore this, we compared volatile emission of *Brassica nigra* plants in response to single or dual attack on their inflorescences. Additionally, we recorded flower visitation by pollinators and the attraction of parasitoids in the greenhouse and/or field. *Brassica nigra* were exposed in the flowering stage to one or two of the following three attackers: *Brevicoryne brassicae* aphids, *Pieris brassicae* caterpillars, and *Xanthomonas campestris* pv. *raphani* bacteria. We found that single attack by caterpillars, and dual attack by caterpillars plus aphids, induced the strongest changes in plant volatile emission. The caterpillars’ parasitoid *C. glomerata* did not exhibit preference for plants exposed to caterpillars only vs. plants exposed to caterpillars plus aphids or plus bacteria. However, the composition of the pollinator community associated with flowers of *B. nigra* was affected by plant exposure to the attackers, but the total number of pollinators visiting the plants did not change upon attack. We conclude that, when *B. nigra* were exposed to single or dual attack on their inflorescences, the plants maintained interactions with natural enemies of the insect attackers and with pollinators. We discuss how chemical diversity may contribute to plant resilience upon attack.

**Supplementary Information:**

The online version contains supplementary material available at (10.1007/s10886-020-01239-6).

## Introduction

Outcrossing plants in the flowering stage need to protect themselves from attack while maintaining pollination in order to ensure their reproduction. Plant volatile emissions play a key role in these processes by mediating mutualistic interactions with natural enemies of herbivorous insects (indirect resistance) and with pollinators (Dudareva et al., 2006; Junker et al., 2010; Kessler et al., 2011; Lucas-Barbosa, 2016; Muhlemann et al., 2014; Schiestl, 2010; Schiestl et al., 2014). While the attraction of pollinators has an evident direct advantage for the reproductive success of most outcrossing plant species, there is increasing evidence that also indirect resistance can benefit plants (Fritzsche-Hoballah & Turlings, 2001; Gols et al., 2015; Kessler & Baldwin, 2001; Lucas-Barbosa et al., 2017; Schuman et al., 2012; van Loon et al., 2000). The attraction of pollinators likely selects for a reliable blend that can be associated with reward quality and quantity (Schiestl & Johnson, 2013; Wright & Schiestl, 2009). However, changes in volatiles emitted by plants upon attack provide essential cues to insectivores to find herbivorous attackers (Kessler & Baldwin, 2001). Therefore, the attraction of pollinators and natural enemies of herbivores may trade off when plants face attackers while in the flowering stage (Bruinsma et al., 2008; Kessler & Halitschke, 2009; Lucas-Barbosa et al., 2011; Schiestl et al., 2014).

The emission of plant odours can vary qualitatively and quantitatively upon attack (Ponzio et al., 2013; Rostás et al., 2006). The induction of specific signal-transduction pathways in response to insect or pathogen attack generally results in specific changes in plant volatile emission, which can subsequently influence the attraction of natural enemies of the plant attackers (Dicke, 1999; Dicke & Baldwin, 2010; Hilker & Meiners, 2002; Turlings & Erb, 2018). Plants face multiple attackers in nature, and, for example, simultaneous attack by multiple herbivores or herbivores plus plant pathogens can interfere with the attraction of parasitoids to their host (Blubaugh et al., 2018; Kroes et al., 2015; Ponzio et al., 2013; Zhang et al., 2009). Other studies, however, showed parasitoid host-finding behaviour to be more robust than expected when considering changes in volatile blends (Erb et al., 2010; Ponzio et al., 2014; Rostás et al., 2006).

Although indirect resistance of plants in the vegetative stage has been extensively addressed (Stam et al. 2014; Turlings & Erb 2018), studies investigating indirect resistance of plants in the flowering stage are scarce, especially when considering florivores. For example, cowpea flowers damaged by *Maruca vitrata* caterpillars emit volatiles that attract parasitoids of these herbivores (Dannon et al., 2010). Plant response to single attack by a florivore may interfere with plant response to another attacker on inflorescences. Dual attack of inflorescences of *Brassica nigra* by combinations of aphids, caterpillars and bacteria induced increased levels of jasmonates (JAs) in the inflorescences compared to plants exposed to single attack (Chrétien et al., 2018). Moreover, the jasmonate content of inflorescences changed in a specific manner depending on the combination of attackers (Chrétien et al., 2018). Jasmonates are involved in the production of floral scent (Stitz et al., 2014), and changes in floral levels of jasmonates may translate into different volatile emissions, among other traits. Jasmonates can, for example, regulate the production of the volatile (*E*)-α-bergamotene, which is known to mediate indirect resistance of *Nicotiana attenuata* (Li et al., 2017a). Interference with the attraction of carnivores upon multiple attack could have strong negative impacts considering the direct damage they inflict on flowers.

Herbivore-induced plant traits that mediate indirect resistance of plants can also influence pollinator behaviour, and consequently, affect the pollination success of a plant attacked in the flowering stage (Kessler et al., 2011; Krupnick et al., 1999; Lehtila & Strauss, 1997; Lucas-Barbosa et al., 2011). Among those traits, herbivore-induced plant volatiles (HIPVs) seem to be important type of cues (Lucas-Barbosa et al., 2011; Schiestl et al., 2014). For example, florivory by the parsnip webworm on wild parsnip induced an increased emission of octyl esters that could be linked to altered pollination success in the field (Zangerl & Berenbaum, 2009). Herbivory on plants in the flowering stage leads to a wide array of consequences for pollinators, ranging from enhanced attraction (Rusman et al., 2018) to deterrence (Bruinsma et al., 2014; Kessler & Halitschke, 2009; Kessler et al., 2011), although herbivory sometimes does not affect pollinator attraction (Pareja et al., 2012). Herbivory can also lead to changes in the time a pollinator spends on a flower, or the number of flower visits by a pollinator (Bruinsma et al., 2014; Lucas-Barbosa et al., 2013). Such effects of herbivory on pollinator recruitment, and the consequences for seed production by the plants, are dependent on the insect species (Rusman et al., 2018), and can be specific to the feeding guild of the herbivorous insect (Rusman et al., 2018), and to the feeding site on the plant (Kessler & Halitschke, 2009). When considering florivores, their feeding damage can directly alter flower numbers and traits, and indirectly induce changes in flower traits via inducible responses in plants (Irwin & Adler, 2006; McCall & Irwin, 2006; Rusman et al., 2019b; Zangerl & Berenbaum, 2009).

The conflict between maintaining floral traits attractive to pollinators while changing traits that attract carnivores may result in a trade-off between indirect resistance and reproduction (Lucas-Barbosa, 2016; Lucas-Barbosa et al., 2011; McCall & Irwin, 2006). *Sinapis alba*, for instance, maintains interactions with both pollinators and parasitoids upon single attack to leaves by aphids (Pareja et al., 2012). *Brassica rapa,* however*,* prioritizes the recruitment of pollinators over the recruitment of parasitoids upon folivory by caterpillars (Desurmont et al., 2015; Schiestl et al., 2014). To understand how herbivory influences pollinators and parasitoids, assessing the effects of herbivory on their attraction to herbivore-infested plants is needed. To date, little is known about the effects of attack of inflorescences on the attraction of parasitoids as well as pollinators in the same system (Lucas-Barbosa, 2016; Pareja et al., 2012; Schiestl et al., 2014). The aim of this study was to explore whether and how multiple attack by an aphid and a caterpillar that mostly florivorous, and by a phytopathogenic bacterium, affects the recruitment of pollinators and natural enemies by a plant in the flowering stage. We investigated the role of plant VOCs as a potential mediator of these two mutualistic interactions. To address this question, we analysed volatiles from the headspace of flowering *B. nigra* plants that had been exposed to single or dual attack, and investigated the behavioural responses of parasitoids and pollinators in greenhouse experiments and in the field. To assess the consequences for plant fitness, we quantified seed set of the plants in the field.

## Methods and Materials

### Study System

The black mustard *Brassica nigra* (Brassicales: Brassicaceae) is a common native plant in The Netherlands that grows in dense patches. This annual species relies on pollinating insects for reproduction (Conner & Neumeier, 1995), although some selfing can occur as well (Lucas-Barbosa et al., 2017; Lucas-Barbosa et al., 2013). Pollinators of *B. nigra* belong to different insect orders, especially the Hymenoptera and Diptera, but also the Lepidoptera. *Brassica nigra* is commonly colonized by the cabbage aphid *Brevicoryne brassicae* (Hemiptera: Aphididae), which is a phloem feeder specialized on brassicaceous plants. This aphid species develops large colonies on inflorescences of *B. nigra*, whereas the development on leaves is limited (Rusman et al., 2019a). The main parasitoid of *B. brassicae* is the solitary wasp *Diaeretiella rapae* (Hymenoptera: Braconidae) (Bahana & Karuhize, 1986; Hafez, 1961; Vaughn et al., 1996) that preferably oviposits in late-instar *B. brassicae* nymphs (Hafez, 1961), and prefers flower-feeding aphids to leaf-feeding aphids (LTS Chrétien, pers. obs.). The gregarious caterpillars of *Pieris brassicae* (Lepidoptera: Pieridae) are specialist herbivores of brassicaceous plants and use *B. nigra* as one of their host plants (Feltwell, 1982). The butterflies lay eggs on leaves (Lucas-Barbosa et al., 2014) and upon hatching, the first-instar (L1) larvae feed on these leaves. The second instar (L2) larvae migrate to the inflorescence and become exclusively florivorous (Lucas-Barbosa et al., 2013). In The Netherlands, caterpillars of *P. brassicae* are often parasitized by the gregarious parasitoid *Cotesia glomerata* (Hymenoptera: Braconidae) which lays a few dozens of eggs in first or second-instar caterpillars of *P. brassicae* (Karowe & Schoonhoven, 1992; Mattiacci & Dicke, 1995). Bacteria, such as *Xanthomonas campestris* pathovar *raphani* (*Xcr*), can infect *B. nigra*. Xcr causes so-called leaf spot disease that mainly affects plants in the Brassicaceae but rarely kills the plants (Machmud, 1982; Vicente et al., 2006). The bacteria can be found in seeds of plants initially infected on the leaves (Machmud, 1982). Xcr causes 1–3 mm large necrotic spots on the infected leaf (Machmud, 1982) and *B nigra* shows relatively high resistance to Xcr (McCulloch, 1929; Ponzio, 2016; Ponzio et al., 2016; Vicente et al., 2006).

### Plant Culture

We grew plants from seeds collected from 25 maternal *B. nigra* plants. Similar quantities of seeds from these 25 maternal plants were pooled and carefully mixed. The maternal plants originated from plants of accession CGN06619 (Center for Genetic Ressources, Wageningen), which had been exposed to open pollination in the experimental farm of Wageningen University for several years.

Experimental plants were grown in pots (Ø17 cm – 2 L content) filled with a 1:1 (*v*/v) mix of sand and potting soil (Lentse Potgrond, Lent, The Netherlands). Plants for the greenhouse experiments were grown in greenhouse compartments (22 ± 2 °C, 60–70% r.h, 16 L:8D), and infested when the first flowers had just opened. Plants for the field experiment were sown in a greenhouse, and seedlings (3–4 leaves) were transferred outdoors to develop in an area protected by insect screen. Field plants were infested within five days after the first flowers opened. *Brassica nigra* plants in full bloom had several hundreds of open flowers.

### Insect and Bacterial Cultures

*Brevicoryne brassicae* aphids and *P. brassicae* caterpillars were reared on Brussels sprout plants (*Brassica oleracea* variety *gemmifera*) in a greenhouse compartment (22 ± 2 °C, 50–70% r.h., 16 L:8D). *Pieris brassicae* butterflies were provided with honey solution from organic production (10%, Melvita, Weide & Veldbloemen) as food, and were kept in a greenhouse compartment (25 ± 2 °C, 50–70% r.h., 16 L:8D). *Diaeretiella rapae* was reared in a climate cabinet (25 ± 1 °C, 16 L:8D) and *C. glomerata* was reared in a greenhouse compartment (22 ± 2 °C, 50–70% r.h., 16 L:8D). Adult parasitoids were provided with honey from organic production and water.

Xcr was obtained from Utrecht University, the Netherlands (Ponzio et al., 2014). The bacteria were cultured in a liquid artificial medium (8 g L^−1^ of Difco™: beef extract 3.0 g L^−1^ and peptone 5.0 g L^−1^, BD Diagnostics, New Jersey, USA) kept at 28 °C under gentle shaking at 170 rpm for 21 ± 1 h. The liquid medium with bacterial cells was then centrifuged twice for 10 min at 4080 rotations per min (rpm) and after each centrifugation the pellet containing the bacterial cells was re-suspended in buffer (10 mM M_g_SO_4_). We estimated the concentration of the inoculum by measuring the light absorbance at 600 nm and adjusted the concentration of the final inoculum to 10^9^ cells mL^−1^ by diluting in buffer (10 mM M_g_SO_4_).

### Plant Treatments

Plants were exposed to buffer, single attack by either *B. brassicae*, *P. brassicae*, or Xcr, or to dual attack by combinations of these attackers when the first flowers opened. To infest the plants with *B. brassicae*, five young adult females were gently placed on a bract (inflorescence leaf) at the base of the inflorescence. The aphids dispersed within a few hours, mainly to the flower stalk, where they multiplied and formed colonies. For the infestation with *P. brassicae*, plants were exposed to mated female butterflies that were allowed to oviposit. We kept a cluster of 30 eggs on the plants and gently removed any surplus of eggs. To infect plants with bacteria, we soaked a 2 × 2 cm piece of cotton wool with 500 μL of the bacterium inoculum (10^9^ cells mL^−1^ in buffer) that we placed on the underside of a bract and maintained for 4 h by a soft clip; control plants (Buffer) were clipped with cotton wool soaked in buffer solution only (10 mM M_g_SO_4_) as described previously (Chrétien et al., 2018). Plants exposed to single or dual attack with the insects *B. brassicae* and/or *P. brassicae* were also clipped with cotton wool with buffer solution to control for a possible effect of buffer and clipping on plant responses. Plants treated with dual attack were simultaneously exposed to two out of the three attackers, and a bract never received more than one treatment. We allowed the aphids and caterpillars to disperse freely over the plant, and focused on the systemic response of plants to attack.

Caterpillars hatched 5 days after oviposition in the greenhouse. To ensure that flowers were damaged for at least one day prior to volatile collection and parasitisation in the greenhouse (on day 8), 50% of the caterpillars were transferred to the inflorescence when they had not yet moved there by themselves on day 7; subsequently, cotton wool was placed around the stem as a barrier between the leaves and the inflorescence. Between 26 and 30 caterpillars generally survived until day 7, and there were generally 3 to 6 aphid colonies on the plants, mostly settled on the inflorescence. Caterpillars hatched between 8 to 16 days after oviposition in the field, and insect densities in the field are further described in the Field Experiment paragraph of the Methods and Materials section.

### Effect of Single and Dual Attack on Volatile Emission of *Brassica nigra* at the Flowering Stage

To investigate whether plant odours are influenced by plant exposure to single versus dual attack, we collected volatiles from the headspace of aboveground parts of the plants after eight days of exposure to one of eight treatments: 1) *B. brassicae*, 2) *P. brassicae*, 3) Xcr, 4) *P. brassicae* plus *B. brassicae*, 5) *P. brassicae* plus Xcr, 6) *B. brassicae* plus Xcr. Plants were watered with 50 mL each, 1 h before the experiment. All insects were removed from the plant just prior to volatile collection.

Volatiles were collected by enclosing the aboveground parts of the plant in an oven bag (Toppits® Brat-Schlauch, polyester; 32 × 32 × 70 cm; Toppits, Minden, Germany). Filtered synthetic air was then flushed into the oven bag at a rate of 300 mL min^−1^ (224-PCMTX*, air-sampling pump Deluxe equipped with an inlet protection filter, Dorset, UK) through a Teflon tube. The air that passed through the bag was then sucked out through a second Teflon tube at a flow rate of 200 mL min^−1^ and led through a metal tube filled with 90 mg of Tenax TA 25/30 mesh (Grace-Alltech). Both Teflon tubes were inserted in the top of the oven bags through an opening that was then closed tightly. The volatile collection lasted for 1.5 h. Oven bags were discarded after use. Volatiles were collected in a greenhouse compartment (25 ± 2 °C, 50–70% r.h., 16 L:8D).

Volatiles were analysed by a gas chromatograph coupled to a mass spectrometer (Thermo Fisher Scientific, Waltham, MA, USA). Plant volatiles were desorbed from the Tenax using a thermodesorption unit (Ultra 50:50, Markes, Llantrisant, UK) that heated the samples from 25 °C to 250 °C (5 min hold) at a rate of 60 °C min^−1^. The released compounds were focused in a cold trap (ID 1.80 mm) at 0 °C that was filled with Tenax and charcoal. The volatiles were transferred in splitless mode to the analytical column (30 m × 0.25 mm ID, 1 μm film thickness, DB-5, Phenomenex, Torrence, CA, USA) by flash heating the cold trap at 40 °C sec^−1^ to 280 °C (10 min hold). The temperature program of the oven started at 40 °C and immediately rose to 280 °C (4 min hold) at a rate of 5 °C min^−1^. Electron impact ionization at 70 eV was used to ionize the column effluent. Mass scanning was carried out from *m/z* 35 to 300 with 4.70 scans sec^−1^. Compounds were identified by comparing the mass spectra with the one of Wiley libraries, NIST and the Wageningen Mass Spectral Database of Natural Products. Identified compounds (listed in Table S1) were confirmed based on retention index using the literature (Adams, 1995).

Peak area was calculated based on total ion chromatograms (TIC) or selected-ion chromatograms (SIC). The SIC integration technique has a better resolution than the TIC technique, therefore, SIC data were used to analyse the effect of treatment on the composition of the volatile blend. Ions were selected based on specificity and mass; their m/z values are listed in Table S1. The TIC technique allows to cumulate peak area of the eluted peaks of a chromatogram and, thus, was used to calculate total volatile emission of plants. Results of this study are based on plant compounds that were detected in at least 50% of the replicates of one of the treatments, and whose peak area was 3.5-fold higher than in background samples (volatiles collected from empty oven bags in which no plant was present) for peaks that were integrated based on TIC, or five-fold higher for peaks that were integrated based on SIC. Peak area of individual compounds was divided by fresh biomass of the aboveground part of the plant for standardization. Plant fresh biomass was measured as soon as the volatile collection was completed: the plant stem was cut at ground level, and the aboveground part was immediately weighed.

Changes in the composition of the volatile blend were analysed with a Projection to Latent Structures - Discriminant Analysis (PLS-DA), with treatment set as the grouping factor. PLS-DA was based on all compounds quantified with the SIC method as described above, to reflect the actual blend emitted by the plant. We tested whether plant exposure to attackers had an effect on the total emission of volatiles using TIC data, with a *Kruskal-*Wallis test and a 0.05 significance level, followed by a *Dunn-Bonferroni post-hoc* test to analyse differences between treatments. We had four to seven plant replicates per treatment.

### Effect of Single and Dual Attack on the Parasitization of *Brassica nigra*’s Attackers by Parasitoid Wasps - Greenhouse Experiments

To investigate whether dual attack affects indirect plant resistance, we assessed the preference of the caterpillar parasitoid *C. glomerata* and of the aphid parasitoid *D. rapae* for plants exposed to dual attack by the host and a non-host compared with plants exposed to single attack by the host. We recorded the plant on which the parasitoid landed first and the plant that was preferred for oviposition in the following two-choice situations: 1) *P. brassicae* vs. *P. brassicae* plus *B. brassicae*, 2) *P. brassicae* vs. *P. brassicae* plus *Xcr* for *C. glomerata*, and 1) *B. brassicae* vs. *B. brassicae* plus *P. brassicae*, 2) *B. brassicae* vs. *B. brassicae* plus *Xcr* for *D. rapae*. Plants were used in the experiments after 8 days of exposure to the treatments. Whenever a plant was inoculated with Xcr, the other plant of the pair was clipped with buffer-soaked cotton wool.

#### *Cotesia glomerata*

Pairs of plants were placed 70 cm apart on a T-shaped platform inside a flight chamber made of gauze (293 × 200 × 230 cm) in a greenhouse compartment (25 ± 2 °C, 50–70% r.h., 16 L:8D). Individual wasps were released at the base of the platform, 90 cm away from the plants. Each wasp was given 10 min to locate a host and we recorded on which of the two plants the wasp first landed and parasitized caterpillars. An observation was stopped as soon as the wasp oviposited in a caterpillar, because *C. glomerata* generally oviposits in all caterpillars of a clutch (Wiskerke & Vet, 1994). When a wasp did not land on a plant within five minutes, the wasp was removed from the flight chamber and this was recorded as non-response. The position of the plants was swapped after every three wasps tested to compensate for possible positional bias. Each female wasp was only tested once, and a maximum of 15 wasps were tested per individual pair of plants. Among those 15 wasps, three to ten wasps responded by flying to a pair of plants and landing on one of them, whereas two to seven wasps subsequently responded with oviposition. When only one wasp responded, that plant pair was excluded from the analysis. All behavioural observations were carried out in the afternoons. Six to eight pairs of plants were used per combination of treatments.

#### *Diaeretiella rapae*

A pair of plants was placed in an igloo mesh tent (70 × 73 × 105 cm) in a greenhouse compartment (22 ± 2 °C, 60–70% r.h., 16 L:8D). One female parasitoid (3-to-6-days old) was released per igloo tent and left with the plants for 20 h. Wasps were given 15 min to accommodate to the new environment, and the location of the wasp (on the tent, on a plant) was recorded after 15 min, 1 h, and 2 h following the release. The plant on which the wasp was first recorded was considered to be the plant that was preferred by the wasp; we assumed that this proxy represented the wasp’s preference because 95% of the wasps stayed on the same plant during the 2 h of recording. After 20 h, the wasp was removed from the cage and the plants were moved to a different greenhouse compartment until aphid mummies developed (25 ± 2 °C, 50–70% r.h., 16 L:8D). The number of mummies was recorded at 7 ± 1 d after the release of the wasp, and we used the highest total number of mummies to determine which plant was preferred for oviposition. Only in one case both plants had exactly the same number of mummies, and no preference was recorded for this pair. We had 18 to 20 pairs of plants per combination of treatments.

#### Statistical Analyses

For the data on *C. glomerata* preference, the effect of plant pair on wasp first landing and oviposition choice was first tested for each combination of treatment using a Generalized Linear Model (GLM) based on a binomial distribution with logit as a link function, and a 0.05 significance level. No effect of plant pair was detected, and wasp choice was then tested regardless of the plant pair. Thus, landing and oviposition preference of both *C. glomerata* and *D. rapae* was tested with a binomial test, with a probability of 0.50 for a wasp to go to one of the plants. The effect of treatment on the number of mummies per plant was tested with a *paired Student’s t test* at the 0.05 significance level; requirements of normal distribution of the data and equal variances were met.

### Effect of Single and Dual Attack on the Parasitization of the Insect Attackers and on the Visitation of Flowers of *Brassica nigra* by Pollinators - Field Experiments

#### Field Layout

**Fig. 1:**
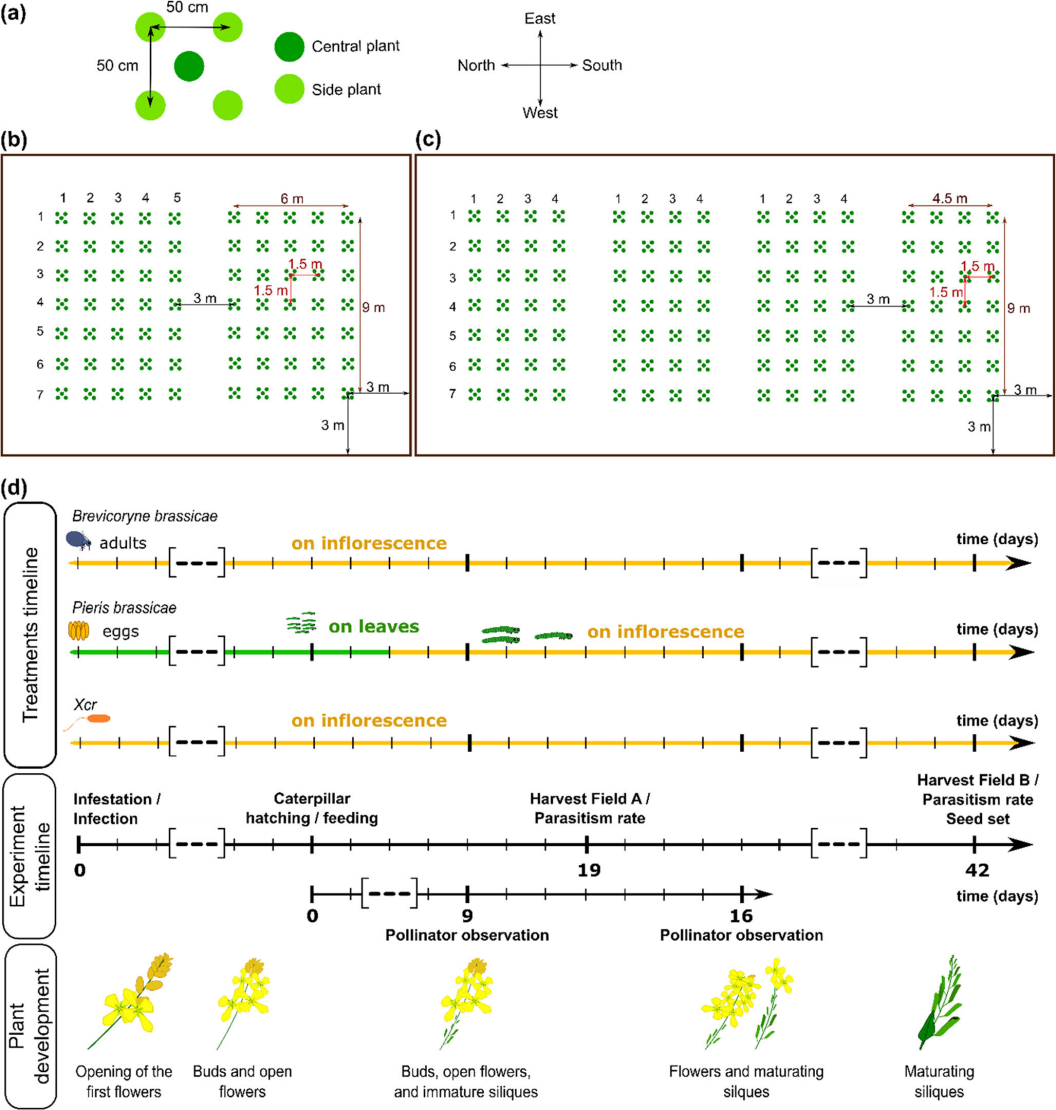
Schematic representation of the layout of the common garden experiment and timeline of the recordings and of the treatments. The compass indicates the orientation of the plots and of the two fields. **(a)** A plot (50 cm × 50 cm) consists of one central plant (dark green) and four side plants (light green). **(b)** Field A consisted of two blocks of 9 m × 6 m, each composed of 35 plots organized in seven rows and five columns. **(c)** Field B consisted of four blocks of 9 m × 4.5 m, each composed of 28 plots organized in seven rows and four columns. (**b, c**) Blocks were 3 m apart, and within a block, central plants of each plot were 1.5 m apart. A fence (brown line) was placed around the fields, 3 m apart from the plots. **(d)** *Brassica nigra* plants were infested with either 5 *Brevicoryne brassicae* aphids or 30 eggs of *Pieris brassicae*, or infected with *Xanthomonas campestris* pv. *raphani* (Xcr). Dual attack consisted of simultaneous attack by two of these organisms. Caterpillars hatched from eggs from 9 to 16 days after infestation, and plants were harvested 19 days after infestation (Field A) to measure parasitism rates and 42 days after infestation (Field B) to measure parasitism rates and seed set. Pollinator visitations were recorded at 9 and 16 days after caterpillars had hatched and fed from the plants. Flower status shows the development of the reproductive parts of the plant over the field experiments

To test whether dual attack to flowering *B. nigra* affects visitation by pollinators and parasitism or predation of insect herbivores in the field, we set up common-garden experiments in which plots of *B. nigra* plants were exposed to one out of seven treatments: 1) *B. brassicae*, 2) *P. brassicae*, 3) Xcr, 4) *P. brassicae* plus *B. brassicae*, 5) *P. brassicae* plus Xcr, 6) *B. brassicae* plus Xcr, and 7) buffer (control). Each plot (50 cm × 50 cm) consisted of five plants (Fig. 1a). Following the design by Lucas-Barbosa et al. (2013), plants were infested/infected in the field directly after transplantation, and only the central plant of a treated plot was originally exposed to one or two of the three attackers. Insect attackers dispersed through the plot and colonized the side plants of a given plot on average within 7 days after infestation for aphids, and about 10 days after hatching for caterpillars. We ensured that the central plant of the plot hosted at least 30 eggs and 15 neonate caterpillars, and at least two colony-founding aphids. Indeed, if fewer than 50% of the caterpillars hatched from the eggs (i.e. < 15 caterpillars), we added neonate caterpillars from the laboratory culture to reach 15 caterpillars. Similarly, when fewer than two aphid colonies were found per plot, we added six adult female aphids from our laboratory culture to develop new colonies. In the field for pollinator observation (field B), we recorded the number of *P. brassicae* caterpillars on plants of plots that received *P. brassicae* eggs as treatment; caterpillars were counted every seven days from the day they hatched. Caterpillar density progressively decreased from egg hatching to pupation due to predation and disease, as has been previously observed (Lucas-Barbosa et al., 2014; Lucas-Barbosa et al., 2013). We counted on average ten caterpillars (1st and 2nd instar) per plot seven days after hatching, four caterpillars (3rd and 4th instar) per plot 14 d after hatching, and only one caterpillar (5th instar – last intsar) was left in the field 21 d after hatching. Densities of *B. brassicae* aphids were also estimated at these same time points, in plots that received aphids as treatment. Densities overall increased from day 7 to day 21: plants at day 7 had from a few tens of aphids to ten colonies of 200–300 aphids; plants at day 21 had from ten colonies of 100–300 aphids to 25 of such colonies, and on rare occasions, hosting a colony of 1000–4000 aphids.

We designed two experimental fields, A and B: in field A, we investigated the effects of dual attack on parasitization of *B. brassicae* and *P. brassicae* at 19 days after infestation/infection (Fig. 1b); in field B, we investigated the effects of dual attack on parasitization of *B. brassicae* and *P. brassicae* at 42 days after infestation/infection, on flower visitation by pollinators, and total seed production (Fig. 1c). Layout of field A (Fig. 1b) consisted of two blocks of 9 m × 6 m each consisting of 35 plots organized in seven rows and five columns. Fourteen plots were transplanted to the field on each day – two plots for each of the seven treatments – within five consecutive days (between June 3rd 2015 to June 7th 2015). Plants were harvested after 19 days of exposure to the treatments (between June 22nd 2015 and June 26th 2015) to measure parasitism upon *B. brassicae* and *P. brassicae*. We had 10 replicates (plots) per treatment. Layout of field B (Fig. 1c) consisted of four blocks of 9 m × 4.5 m, each composed of 28 plots organized in seven rows and four columns. Fourteen plots were transplanted to the field on each day – two plots for each of the seven treatments – within eight consecutive days (between May 19th 2015 and May 26th 2015). Plants were harvested 42 days after infestation/infection (between June 30th 2015 and July 7th 2015) to quantify total seed set; parasitization of *B. brassicae* and *P. brassicae* were monitored as well. We had 16 replicates (plots) per treatment. In both fields A and B, blocks were 3 m apart, and within a block, the central plants of plots were 1.5 m apart. Treatments were assigned to plots according to a Latin square design to control for environmental bias. We also ensured that plants of the same treatment that were infested/infected on the same day, were never planted in the same column or row. A fence was placed around each field, 3 m from the nearest plots, to protect the fields from relatively larger herbivores such as rabbits. The ground area around the plots was regularly weeded.

#### Parasitization of Aphids and Caterpillars

The number of aphid mummies on all central plants and on two side plants of a given plot was counted at 19 days after infestation in field A and 42 days after infestation in field B. Plants were first harvested and then living aphids were gently brushed off the plants to uncover all mummies for counting. *Brevicoryne brassicae* are the main aphids developing on *B. nigra* in our field, and mummies are assumed to mainly belong to this species. Numbers of mummies per plant were averaged at the plot level. Numbers of mummies at day 19 were analysed with a Generalized Linear Mixed Model (GLMM) based on a negative binomial distribution and log as link function. Numbers of mummies at day 42 were normally distributed, and variances could be assumed as equal, therefore these data were analysed with a Linear Mixed Model (LMM). In both cases, the main effect of treatment was set as fixed factor and intercept was included, we added the planting day as a random factor. We used a significance level of 0.05. Parasitization of *P. brassicae* was estimated by dissecting caterpillars to check for the presence or absence of parasitoid eggs in L1/L2 caterpillars. Caterpillars were collected from the plants of field B after 19 days since infestation of the plants with butterfly eggs.

#### Pollinator Visitation to *Brassica nigra* Exposed to Multiple Attack in the Field

We recorded pollinator visitation to plots of treated and control plants of *B. nigra* after 9 d and 16 d of caterpillar feeding. At day 9, caterpillars were at the L2/L3 stage and had been feeding from flowers for 1–2 days; plants mainly had flowers and buds. At day 16, caterpillars were in the L5 stage and had been feeding from flowers for 8–9 days; plants had flowers and unripe siliques. The two observation time points were determined based on the number of days that plants were exposed to caterpillar feeding because caterpillars hatched from the eggs within a time window of 12 to 16 days after egg deposition, irrespective of the treatments. Plots with no caterpillars as treatment were observed based on the caterpillar-treated plots of the same planting date.

Each plot was observed for 10 min, using a handheld computer (Psion Workabout Pro TM3, London, UK) and the Observer software (version 10, Noldus Information Technology b.v., Wageningen, The Netherlands; Noldus 1991). Observations were performed between 9 am and 6 pm, when the wind condition was low and there was no rain. The temperature ranged from about 15 to 25 °C. We randomized the order in which plots were observed each day. However, external factors did not always allow us to monitor two replicates of all seven treatments each day. When this happened, we observed at least one replicate of each treatment per day.

To investigate the effect of single and dual attack on the attractiveness of flowers, we recorded the number and identity of pollinators visiting a given plot of plants. Pollinator identity was classified into four groups: 1) bees, 2) bumblebees, 3) flies, 4) butterflies, and we identified the most abundant flower visitors to the species level. We distinguished honey bees (*Apis melifera*) and solitary bees within “bees”. “Bumblebees” included *Bombus lapidarius*, *Bombus terrestris*, and other bumblebees visiting the flowers. “Flies” included the syrphid *Eristalis tenax* and other fly species. “Butterflies” included *Pieris rapae* and other butterflies visiting the flowers. We also recorded the time spent between the moment a pollinator first arrived to the flowers of the plot until the moment it left the plot’s plants, and we counted the number of flowers visited over this time period. Thus, we could calculate the time a pollinator spent on average per flower. Once the visitor had left, we would start following a new one, and repeated this over the 10 min of observation. We cannot exclude the possibility that the same pollinator returned to the plot after having left, and if so, its visit was recorded as a new visitation.

Effect of treatment on the total number of pollinators was analysed with an LMM, data were normally distributed and met the assumption of equal variances. We used a GLMM based on a normal distribution with identity as a link function to test the effect of treatments on time spent per flower by honeybees and flies, and for the number of flowers that honeybees visited. For the number of flowers that flies visited, we used a GLMM based on a negative binomial distribution, with logit as a link function. Data of one individual honeybee (Aphid plus bacteria treatment, day 9) was excluded; this insect spent 10 times more time on a flower than average and was considered as an outlier. For all LMM and GLMM analyses, the main effect of treatment was set as fixed factor and the intercept was included; in addition, the date when the plot was observed (observation date), which was confounded with weather conditions, was set as a random factor. Total number of pollinators at day 9 and day 16 were compared with a *G-test*. Effect of treatment on the assemblage of the pollinator community was tested with a *Chi-square test* at each time point. Results indicated that at least one treatment differed from the expected community. We removed the treatment with the most extreme distribution (*B. brassicae* plus *P. brassicae*) and ran a new *Chi-square test*: no other treatment differed from the expected community composition. The significance level was 0.05 in all cases.

### Effect of Single and Dual Attack on the Seed Set of *Brassica nigra* in the Field

We determined the seed set of *B. nigra* plants in field B after 42 days of exposure to the treatments. Siliques were stored to dry at room temperature in the dark in a farm building (Unifarm, Wageningen University) until seeds were processed and counted. The number of seeds was estimated by dividing the weight of the total number of seeds of a given plant by the weight of 100 seeds of this plant. Seeds were counted for the central plant and the two randomly selected side plants for each plot, and we calculated the average number of seeds produced per plant per plot. In four cases, the central plant had died during the experiment, and in three cases, the seed bag of the central plant could not be identified. Data related to these plots were not included in the analyses.

Effect of treatment was analysed with an LMM. Main effect of treatment was set as fixed factor and intercept was included, the planting date was set as a random factor, and we used a significance level of 0.05.

### Statistical Software and Procedures

We used respectively the default GENMIX, GENLIN, and MIXED procedures of SPSS (IBM Corp., IBM SPSS Statistics for Windows, Versions 24, Armonk, NY: IBM Corp.) to run GLMMs, GLMs, and LMMs. Chi-square tests and G-tests were performed in Excel (version 2016, for Windows, Microsoft® office, Redmond, Washington, USA). PLS-DAs were performed in SIMCA (Umetrics AB, Version 15.0, Umeå, Sweden), and we used the default 7-fold cross-validation (CV) procedure to calculate model fit parameters: the number of significant components, the goodness of fit R^2^X and R^2^Y, and the predictive ability Q^2^Y. R^2^X and R^2^Y represent respectively the percentage of variation explained by the matrix of volatile data (X) and by the matrix of treatments (Y). In poor models, the order of rows in the original data set can affect the value of Q^2^Y (Triba et al., 2015). Thus, we ran each PLS-DA for four datasets with randomly permuted rows, and we display the averaged Q^2^Y value ± standard deviation; models were stable and there was little variation. We used Inkscape 0.92.0 and 0.92.4 to combine figures and make drawings.

## Results

### Effect of Single and Dual Attack on Volatile Emission of *Brassica nigra* at the Flowering Stage

No qualitative differences were recorded when comparing compounds in the volatile blend emitted by aboveground parts of flowering *B. nigra* exposed to the different treatments. We detected, identified, and quantified 59 compounds belonging to seven classes of volatile compounds based on SIC (Table S1): 36 terpenes (26 monoterpenes, 1 homoterpene, 8 sesquiterpenes, 1 homosesquiterpene), 9 aromatic benzenoids and phenylpropanoids, 5 fatty acid or amino acid derivatives, and 6 nitrogen-containing compounds (including glucosinolate derivatives), plus 3 compounds that could not be identified and classified.

**Fig. 2:**
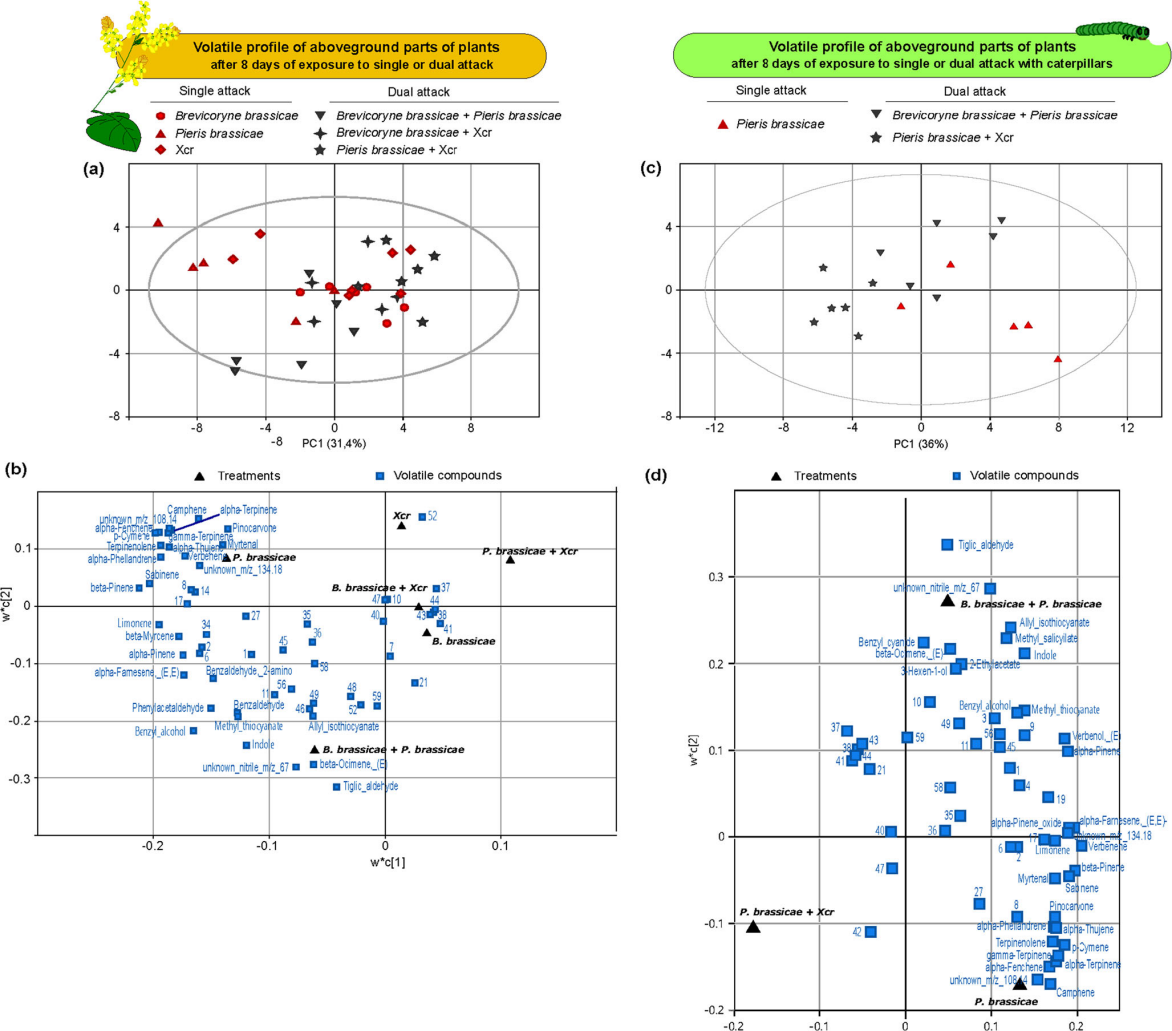
Volatile profiles of aboveground parts of flowering *Brassica nigra* plants exposed to buffer (light grey), single attack (red), and dual attack (dark grey). Projection to Latent Structures - Discriminant Analysis (PLS-DA) based on the quantity of 59 volatile compounds (expressed as peak area /10^9^ g^−1^ of plant fresh biomass) that could be detected and quantified using chromatograms based on single ion chromatograms (SIC) in samples of *B. nigra*. Volatile blends were collected for 1.5 h from aboveground parts of *B. nigra* exposed for 8 days to either: (a, b) single or dual attack by *Brevicoryne brassicae* aphids, *Pieris brassicae* caterpillars*,* and/or *Xanthomonas campestris* pv. *raphani* (Xcr) bacteria, or (c, d) single attack with *Pieris brassicae* caterpillars, dual attack with *Brevicoryne brassicae* aphids plus *P. brassicae* or with *P. brassicae* plus Xcr bacteria. Treatments were set as classes in the PLS-DA. (a, c) Scatter plots show grouping pattern of samples from a same treatment according to the first two principal components (t[1], t[2]). The percentage between brackets indicates the percentage of variation in the data explained by each principal component. The Hotelling’s ellipse confines the confidence region (95%) of the score plot. (b, d) Loading plots show the contribution of each of the volatile compounds’ quantifications to the first two principal components. Compounds with a VIP > 1 are shown with full names. For the other compounds: 1 = 2-phenylethanol; 2 = benzylacetate; 3 = benzaldehyde; 4 = benzaldehyde-2-amino; 6 = methyl-phenyl acetate; 7 = methyl salicylate; 8 = p-anisaldehyde, 9 = phenylacetaldehyde; 10 = (*Z*)-2,6-dimethyl-1,3,5,7-octatetraene; 11 = alloocimene, neo; 14 = α-pinene oxide; 17 = α-terpineol; 19 = β-myrcene; 21 = β-ocimene-epoxide (*E*); 27 = linalool; 34 = verbenol (*E*); 35 = verbenone; 36 = (*E*)-4,8-dimethyl-1,3,7-nonatriene; 37 = 7-α-H-silphiperfol-5-ene; 38 = 7-β-H-silphiperfol-5-ene; 40 = α-farnesene (*Z,E*); 41 = β-caryophyllene (*E*); 42 = presilphiperfol-7-ene; 43 = silphiperfol-5,7(14)-diene; 44 = silphiperfol-6-ene; 45 = 4,8,12-trimethyl-1,3,7,11-tridecatetraene (*E,E*); 46 = 2-ethyl-acetate; 47 = 2-methylbutanoic-acid-methyl-ester; 48 = 3-hexen-1-ol (*Z*); 49 = 3-hexen-1-ol-acetate (*Z*); 52 = benzyl-cyanide; 56 = unknown thiocyanate; 58 = unknown_m/z_119.16; 59 = unknown_m/z_150.17

Treatments led to quantitative changes in the composition of the volatile blend emitted by aboveground parts of *B. nigra* plants exposed to single and simultaneous dual attack by *B. brassicae* aphids, *P. brassicae* caterpillars and Xcr bacteria (Fig. 2). A Principal Latent Structure Discrimination Analysis (PLS-DA) based on the samples of the six treatment combinations resulted in a model with one significant principal component (R^2^X = 0.314, R^2^Y = 0.104, Q^2^ = 0.045 ± SD 0.007). We display here the projection of the data for plant samples over two principal components for visual representation (Fig. 2a). The first principal component (PC1) explains 31.4% of the variation and separates plant samples according to plant exposure to caterpillars. Blends of plants exposed to caterpillars only, and to a lower extent, plants exposed to aphids plus caterpillars, differ from the blends of plants exposed to the other treatments. Fifty percent (29 VOCs) of the VOCs contribute most to the differentiation of the blends (VIP > 1). Most of them are more associated to the blend of plants exposed to caterpillars and to aphids plus caterpillars, indicating that they are emitted at higher rates by these plants compared to plants exposed to single attack with aphids or with bacteria, or to dual attack with the bacteria plus an insect (Fig. 2b, Table S1). The VOCs that contribute most to the separation described are mainly monoterpenes (18), representing 70% of all monoterpenes detected in the blend (Fig. 2b, Table S1). Additionally, four out of six nitrogen-containing VOCs detected in the blend contribute to the separation described above, including glucosinolate derivatives (Fig. 2b, Table S1).

We analyzed in simpler models whether the blend emitted by plants exposed to single attack differed from the blend of plants exposed to dual attack, to further link changes in volatile emission with the behaviour of natural enemies of the insect attackers in single attack and dual attack situations. Therefore, the blend of plants exposed to single attack with *P. brassicae* caterpillars was compared to the blends of plants exposed to dual attack with *P. brassicae* and another attacker, similarly the blend of plants exposed to single attack with *B. brassicae* aphids was compared to blends of plants exposed to dual attack with *B. brassicae* plus another attacker. A PLS-DA based on the samples of plants exposed to attack by *P. brassicae*, *P. brassicae* plus *B. brassicae*, or *P. brassicae* plus Xcr, resulted in a model with one significant principal component (R^2^X = 0.360, R^2^Y = 0.344, Q^2^ = 0.228 ± SD 0.008), and confirms that plants exposed to caterpillars plus Xcr differ from samples of plants exposed to caterpillars and aphids plus caterpillars according to PC1 (36% of the variation, Fig. 2c). However, blends of plants exposed to caterpillars could not be separated from blends of plants exposed to aphids plus caterpillars. VOCs contributing to the separation between blends were mostly the same as in the PLS-DA based on all treatments (VIP > 1), and the difference in blend composition was mainly driven by over 70% of the 26 monoterpenes detected, five out the six nitrogen-containing compounds detected, and three out of the five fatty acid derivatives (Fig. 2d, Table S1). PLS-DA based on VOC emission of plants exposed to single attack by *B. brassicae*, *B. brassicae* plus *P. brassicae*, or *B. brassicae* plus Xcr did not result in a model with a significant PC, indicating that the model could not separate the blends based on their composition.

Differences in the total volatile emission (based on TIC) of *B. nigra* upon attack are similar to those observed for the composition of the VOC blends. Overall, treatment affects total volatile emission (Fig. S1, *Kruskal-Wallis*, chi-square = 14.159, df = 7, *P* = 0.048). Total emission of plants exposed to aphids plus caterpillars significantly differs from total emission of plants exposed to caterpillars plus bacteria.

### Effect of Single and Dual Attack on the Parasitization of *Brassica nigra*’s Attackers by Parasitoid Wasps

**Fig. 3:**
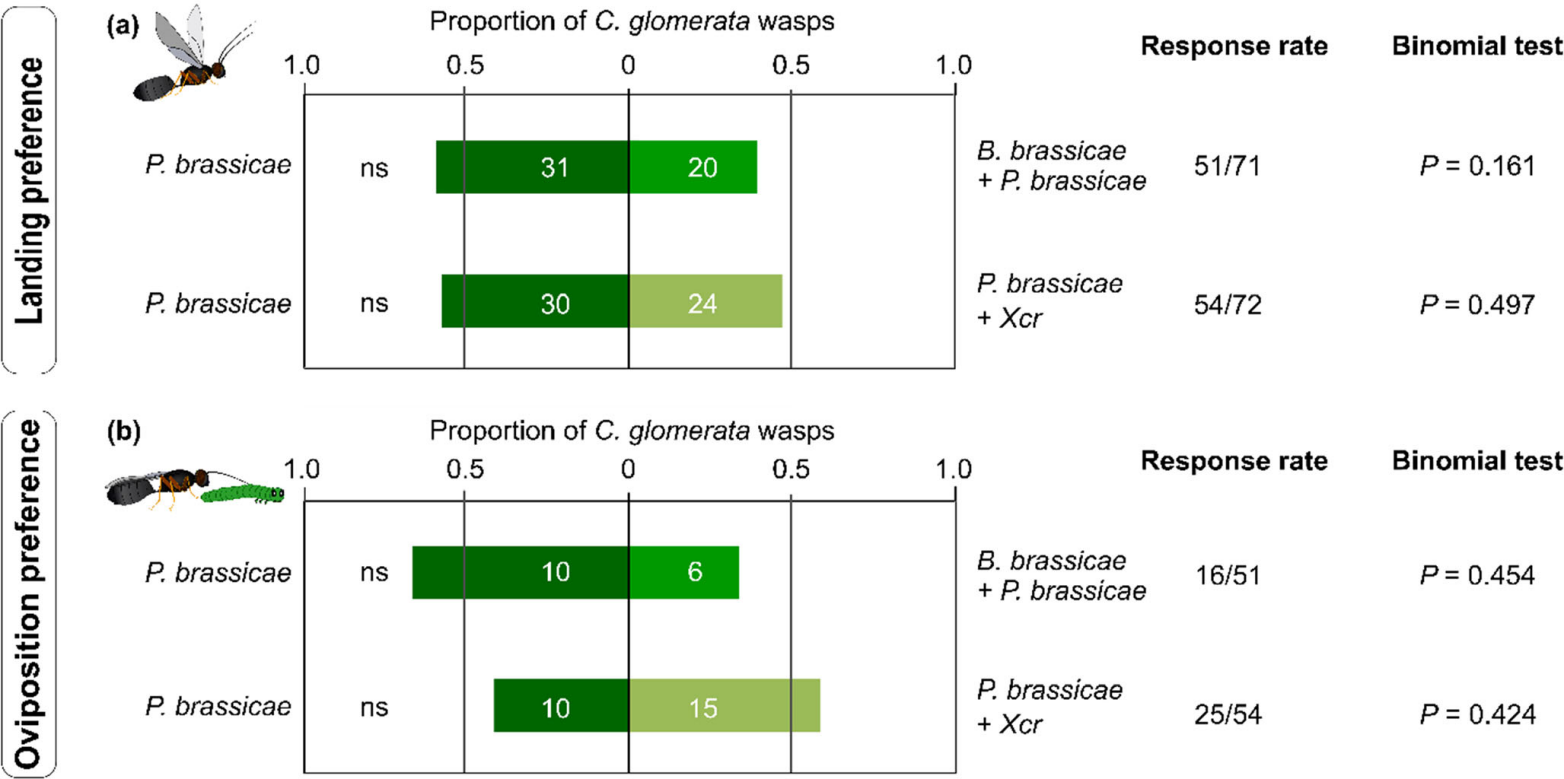
Proportion of *Cotesia glomerata* that landed and oviposited on flowering *Brassica nigra* plants exposed to single attack with caterpillars (host) vs. plants exposed to dual attack with caterpillars (host) plus aphids or plus bacteria (non-hosts). Preference of *C. glomerata* wasps was tested in a two-choice assay in a greenhouse. Plants were either exposed to single attack by *Pieris brassicae* caterpillars, or exposed to dual attack by *Brevicoryne brassicae* aphids plus *P. brassicae* or by *P. brassicae* plus *Xanthomonas campestris* pv. *raphani* (Xcr) bacteria. Plants exposed to single and dual attack were combined two by two in a flight chamber where a *C. glomerata* wasp was released for 10 min. We scored plants on which the wasps landed first (**a**) and plants on which *C. glomerata* first oviposited (**b**). Response rate indicates the number of responding wasps over the number of tested wasps. Proportions were tested using a binomial test, and the significance level was set to α = 0.05

In the greenhouse, dual attack did not influence the first landing and oviposition preference of *C. glomerata* in a two-choice assay where *B. nigra* plants were exposed to the host caterpillars alone vs. plants exposed to hosts plus a non-host that was either aphids or bacteria (Fig. 3a, b). In the field, across all treatments, 97% of the 60 caterpillars recollected were parasitized.

Similarly, dual attack did not influence the first landing and oviposition preference of *D. rapae* in the two-choice assay where *B. nigra* plants were exposed to the host aphid alone vs. plants exposed to hosts plus a non-host that was either caterpillar or bacteria, but numbers of parasitoid responses were low (Fig. S3a). In the common-garden experiment, treatments did not influence the number of aphid mummies, a proxy of aphid parasitization, recorded on plants that were initially exposed to aphids alone or to aphids plus caterpillars or bacteria (Fig. S3b, c).

### Effect of Single and Dual Attack on the Visitation of Flowers of *Brassica nigra* by Pollinators

#### Number of Pollinators

**Fig. 4:**
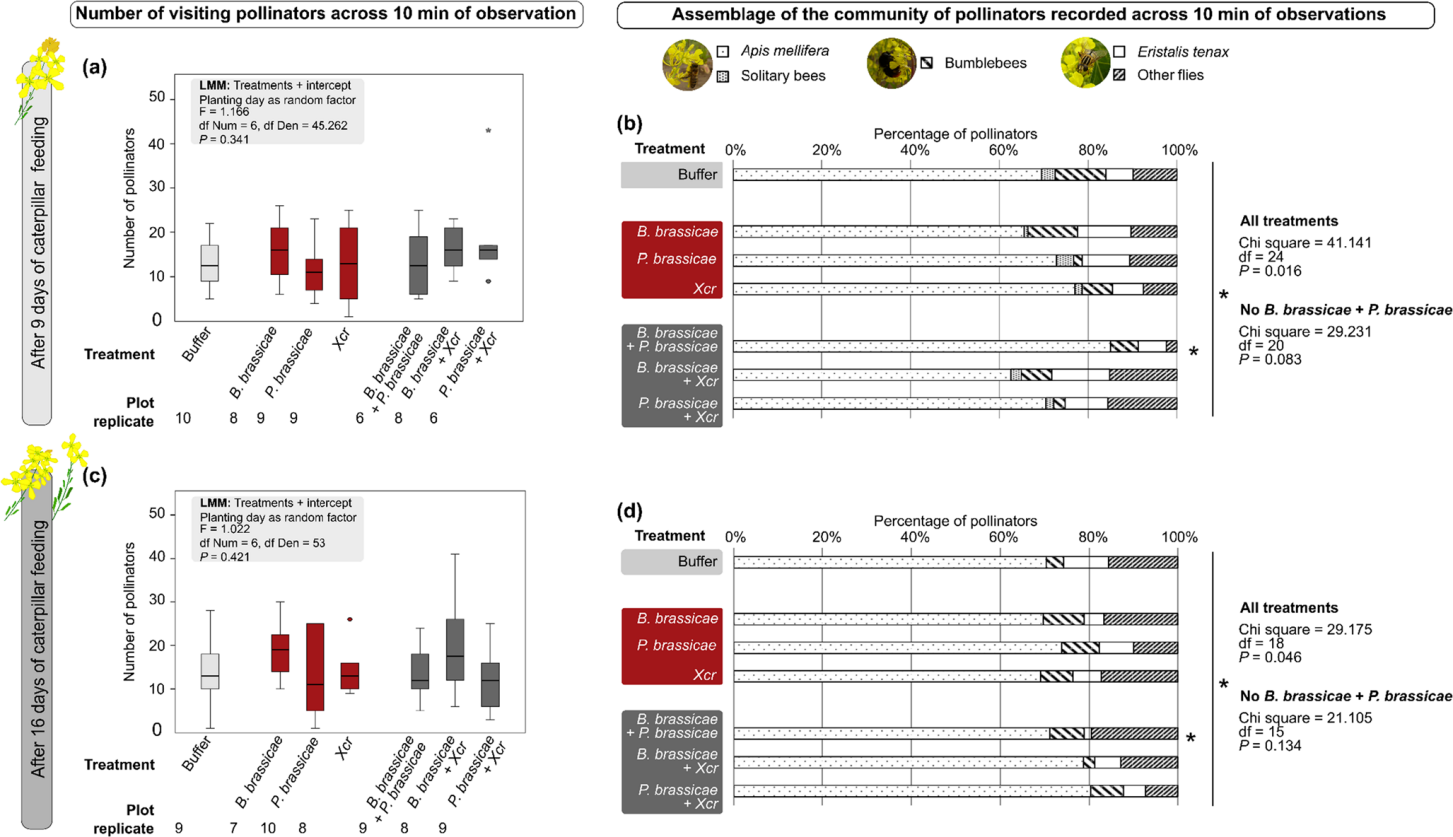
Number of pollinators (median, interquartile range, full range) and assemblage of this community of pollinators (%) visiting plots of *Brassica nigra* exposed to buffer (light grey), single attack (red), and dual attack (dark grey) in the field. Plots of five *B. nigra* plants were observed for 10 min and all pollinators arriving to the plot were counted and recorded as either *Apis mellifera* (honeybee), solitary bees, bumblebees, *Eristalis tenax*, and other flies. Pollinator observation took place after 9 d of caterpillar feeding (**a, b**) and after 16 d (**c, d**). Numbers of pollinators were summed per plot (**a, c**) and the contribution of each type of pollinator (%) to this total community was calculated (**b, d**). Butterflies were excluded as they represented less than 1% of the whole community. Plots were exposed in the field to single attack by either *Brevicoryne brassicae*, *Pieris brassicae,* and/or *Xanthomonas campestris* pv. *raphani* (Xcr), to dual attack by combinations of two of those attackers, or exposed to buffer (control). Outliers are represented by “ ° ” (further than 1.5 x interquartile range). Effect of treatments on total number of pollinators was analysed with a Linear Mixed Model (LMM). Effect of treatments on community assemblage was analysed with a *Chi-square test*. At both time points, results indicated that at least one treatment differed from the expected community. We removed the treatment with te most extreme distribution from the analysis (*B. brassicae* plus *P. brassicae*) and ran a new *Chi-Square*, which showed that no other treatment differed from the expected community. The significance level was set to α = 0.05

Overall, bees were the most abundant pollinators (73.4% at day 9 and 74.1% at day 16), followed by syrphid flies (9.5% on day 9 and 5.4% on day 16), then bumblebees (6.1% on day 9 and day 16) and finally butterflies (0.1% on day 9 and day 16) (Table S2). Similar numbers of pollinators visited the plants on day 9 and day 16 irrespective of the treatments (Table S2, G-test, *P* = 0.168). We recorded 790 pollinators over 56 plots after 9 days of caterpillar feeding, and 905 pollinators over 60 plots after 16 days of caterpillar feeding. Treatments did not affect the number of pollinators visiting the *B. nigra* flowers neither on day 9 nor on day 16 (Fig. 4 a,c).

#### Assemblage of the Pollinator Community

Plant exposure to the attackers had an effect on the assemblage of the community of pollinators at both time points. The pollinator community of plants exposed to dual attack by aphids plus caterpillars particularly differed from the pollinator community of plants exposed to single attack or other combinations of dual attack (Fig. 4b, d, Table S2). At the first time point, this difference seems to be driven by the number of flies visiting plants exposed to dual attack by aphids plus caterpillars (Table S2). About three times fewer flies visited plants attacked by aphids plus caterpillars when compared with plants attacked by caterpillars only or aphids only, and about four times fewer flies visited plants attacked by aphids plus caterpillars compared with plants exposed to other dual-attack treatments (Table S2, Fig. 4b). At the second time point, almost twice as many flies visited plots attacked by aphids plus caterpillars compared with plots where plants were attacked by caterpillars plus bacteria (Table S2, Fig 4d).

#### Time Spent per Flower

The time that honeybees and flies spent per flower at each of the two observation time points was not influenced by the treatments (Fig. S4), and neither was the number of flowers visited in a row by a bee or a fly (Bees, LMM, day 9: F = 0.678, Numerator df = 6, Denominator df = 44.835, *P* = 0.668; day 16: LMM, F = 1.026, Numerator df = 6, Denominator df = 47.680, *P* = 0.420; Flies, GLMM, day 9: F = 1.629, df1 = 6, df2 = 15, *P* = 0.207, day 16: low replication did not allow statistical analyses).

### Effect of Single and Dual Attack on Seed Set of *Brassica nigra* in the Field

**Fig. 5:**
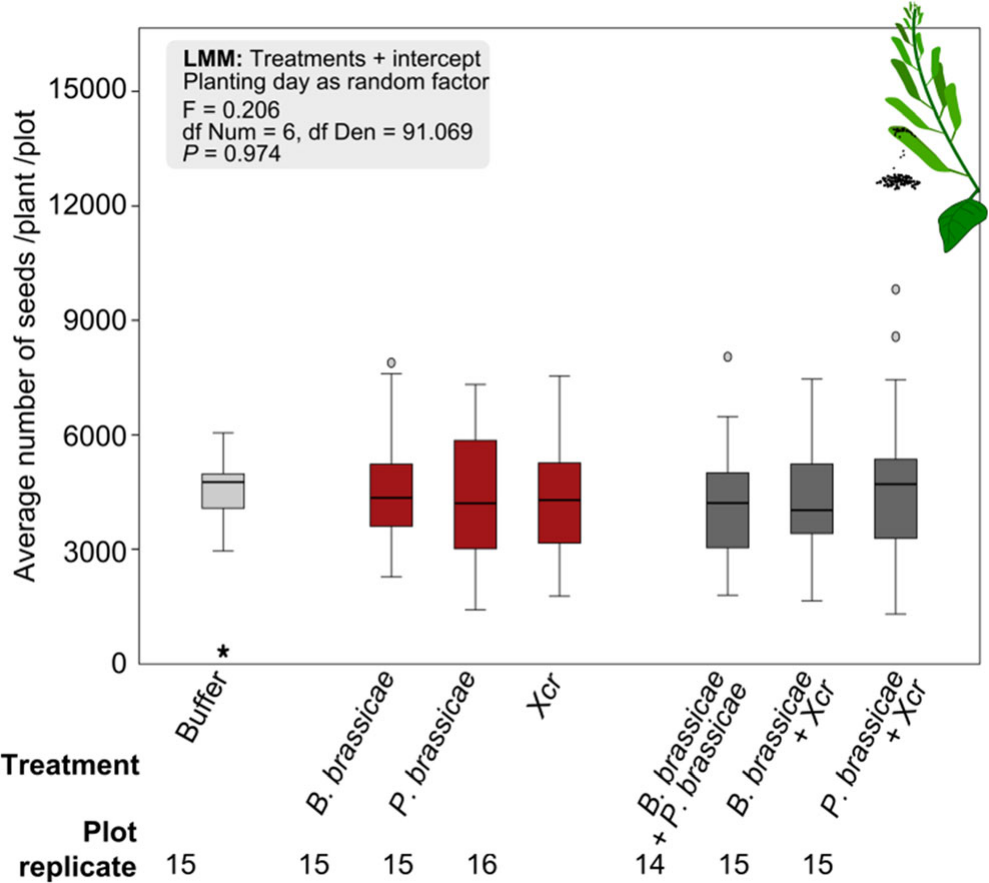
Average number of seeds (median, interquartile range, full range) produced by plants of each plot of *Brassica nigra* plants exposed to buffer (light grey), single attack (red), and dual attack (dark grey) in the field. Plots of *B. nigra* were exposed in the field to single or dual attack by *Brevicoryne brassicae* aphids, *Pieris brassicae* caterpillars*,* and/or *Xanthomonas campestris* pv. *raphani* (Xcr) bacteria, or exposed to buffer (control). After 42 d, seeds were harvested, weighed and their numbers estimated for the central plant and two side plants of each plot; we show the average number of seeds produced per plant per plot. Outliers are represented by “ ° ” (further than 1.5 x interquartile range). Effect of treatments was analysed with a Linear Mixed Model (LMM). The significance level was set to α = 0.05

Plants of plots exposed in the field to an initial attack by *B. brassicae* aphids, *P. brassicae* caterpillars, or Xcr bacteria produced on average similar numbers of seeds as plants of plots exposed to dual combinations of those attackers or to buffer (control plots) (Fig. 5).

## Discussion

Our study shows that flowering *B. nigra* exposed to single attack with three different attackers, or to dual combinations of these, remained attractive to parasitoids and pollinators despite the fact that treatments affected the volatile blend emitted by the plants. Caterpillars, in single attack or dual attack in combination with another attacker, were the main inducers of changes in plant volatile emission, and 50% of the 59 VOCs emitted by flowering *B. nigra* contributed to the changes. Both natural enemies and pollinators rely on several cues, such as olfactory and visual cues, and their interaction with the plant might be affected when the plant cues change upon herbivory. In our study, parasitoid preference was resilient to the changes in volatile emission, and plant exposure to non-host attackers neither affected choices of parasitoids in the greenhouse nor parasitism in the field. Similarly, we found that plant exposure to attackers did not impact the number of pollinators visiting flowers within the time frame of our observations, although attack to the inflorescences of *B. nigra* can alter the composition of the pollinator community. *Brassica nigra* interacts with over 10 different pollinator species from at least three insect orders, and negative effects of induced changes in floral traits to a subset of the community may be buffered by the attraction of other pollinator species. Interacting with diverse mutualists likely supports the maintenance of pollination and indirect resistance upon multiple attack.

Our data show that there were treatment-specific changes in volatile emission when plants were exposed to attack on their inflorescences. Data from our previous study show that inflorescences of *B. nigra* exposed to *P. brassicae*, *B. brassicae* or *Xcr*, or to dual infestations by combinations of these attackers had distinct phytohormonal profiles, and that caterpillars in particular induced the active forms of JA in inflorescence tissues (Chrétien et al., 2018). Effect of treatments on volatile emission seems to be in line with these patterns in phytohormonal induction upon attack. We detected differences in plant volatile emission when comparing plants exposed to either caterpillars only, caterpillars plus aphids, or caterpillars plus bacteria, whereas no difference was detected when comparing volatile emission of plants exposed either to aphids only, aphids plus caterpillars, or aphids plus bacteria. Thus, attack by the caterpillars (alone or in combination with another attacker) seems to drive the strongest changes in plant volatile emission, compared to aphid treatments (alone or in combination with another attacker). Moreover, the volatile blend of plants exposed to caterpillars more specifically differed from the volatile blend of plants exposed to caterpillars plus bacteria. The inducible emission of volatiles by plants in the flowering stage has so far mainly been explored for single attack, and most studies focused on plant responses to folivory (Bruinsma et al., 2014; Kessler et al., 2011; Lucas-Barbosa et al., 2011; Pareja et al., 2012; Schiestl et al., 2014; but see Rusman et al. (2019b) for effect of florivory). The attacker-specific changes in volatile emission observed here suggest that plants in the flowering stage are also able to perceive, recognize and respond to different types of attack on their inflorescences, and JA and its derivatives are the likely orchestrators of plant response to florivory (Chrétien et al., 2018; Li et al., 2018). Besides the role of JA in the induction of HIPVs (Kessler & Baldwin, 2002), an increase in JA levels can also lead to reduced nectar production (Bruinsma et al., 2008). Recent studies have also demonstrated that flower colour and flower shape are as well affected when flowering *B. nigra* plants are exposed to different attackers (Rusman et al., 2019b).

Herbivore-induced plant responses can be specifically perceived and exploited by multiple members of the plant community that can all influence plant fitness, in a negative or positive manner (Kessler & Halitschke, 2007). We found that at both time points, plants exposed to caterpillars plus aphids harboured a different pollinator community than the other treatments; however, the total number of pollinators visiting the plant did not change. Plants may buffer detrimental effects of attack on some of their pollinators by attracting other ones. Such ecological plasticity is especially an option for flowers that are not pollinator limited and interact with a diverse range of pollinators (Lucas-Barbosa, 2016; Rusman et al., 2018). Inflorescences of *B. nigra* recruited on average one to two pollinators per minute, which visited several flowers in a row. This abundance of pollinators probably explains why the total number of pollinators attracted to the plants was not affected by plant exposure to the attackers. Different pollinators harvest different types of rewards and can exploit different flower traits. As a consequence, changes in some flower traits in response to herbivory or pathogen attack may affect only a subset of the pollinator community (Junker et al., 2013; Lucas-Barbosa, 2016; Schiestl & Johnson, 2013). *Brassica nigra* interacted with over 10 species of flower visitors belonging to three different orders: Hymenoptera, Diptera, and Lepidoptera. In our study, most community changes were due to fewer flower visitations by syrphid flies, a pattern that has also been observed by Rusman et al. (2018). If attack results in changes in a flower trait that interfere with pollinator attraction, this may be compensated by an increase in visitation by other types of pollinators. Thus, generalist flowers may benefit from ecological plasticity upon attack.

Despite the chemical changes induced by dual attack in flowering *B. nigra,* the co-occurrence of two distinct attackers did not affect the attraction and oviposition preference of the parasitoids. For plants in the vegetative stage, some studies highlighted HIPV-driven changes in parasitoid behavior in the presence of non-hosts (Dicke et al., 2009; Ponzio et al., 2013). For *B. nigra* in the vegetative stage, however, co-infestation by *P. brassicae* caterpillars with *B. brassicae* aphids*,* eggs of *P. brassicae,* or Xcr bacteria induced changes in the volatile emission of the blend, but the parasitoid *C. glomerata* could still locate its host in two-choice assays with plants exposed to the host caterpillar vs. plants exposed to the host and a non-host (Cusumano et al., 2015; Ponzio et al., 2014). Similarly, *D. rapae* and other aphid parasitoids tend to maintain their ability to locate their host upon multiple attack on *Brassica juncea* (da Silva et al., 2016). Cabbage HIPV blends are complex and changes cannot be linked to parasitoid attraction in a straightforward way (Li et al., 2017b; Ponzio et al., 2014). The HIPV emission that we measured represents the full sampled headspace and is likely broader than the subset of volatiles that is used by the parasitoids (Ponzio et al., 2014). In flowering *B. nigra*, we detected as many as 59 compounds that belong to at least seven classes of compounds; thus, we can consider *B. nigra*’s odour as a complex blend (Dudareva et al., 2006). Among the blend components, 50% contributed most to the separation of the blends upon attack in the multivariate analysis, the others having little or no contribution to the differences between the blends. It is, thus, possible that the subset of HIPVs perceived and used by the parasitoid was little affected upon dual attack. Therefore, complex HIPV blends may provide a certain chemical plasticity to attacked plants.

Plants may benefit from the attraction of a diverse community of specialist and generalist insectivores that can be resilient to changes in plant cues used by insectivores when plants are attacked by multiple organisms. Natural enemies of herbivores are diverse and abundant on brassicaceous plants (Lucas-Barbosa et al., 2017; Lucas-Barbosa et al., 2014; Stam et al., 2018). These insectivores are an important component of the resistance strategy of plants in the flowering stage (Gols et al., 2015; Knauer et al., 2018; Lucas-Barbosa et al., 2017; Lucas-Barbosa et al., 2014). On *B. nigra*, mortality of herbivores on the plants was caused by parasitoids and predators, with few of the caterpillars surviving until the adult stage. Like pollinators, insectivores are known to use a wide array of cues to find their prey or host for oviposition, and these cues can be olfactory, visual, or gustatory (Kessler & Halitschke, 2007; Stam et al., 2014). The diversity of insectivores encountered on aboveground parts of *B. nigra* is presumably large leading to nearly 100% mortality of caterpillars. Insectivores mainly belonged to six orders: Hymenoptera, Diptera, Heteroptera, Coleoptera, Aranea, and Acarina (Lucas-Barbosa et al., 2014), (L.T.S. Chrétien and D. Lucas-Barbosa, pers. obs). Thus, abundance and diversity of natural enemies may provide *B. nigra* with a flexible means to indirectly resist multiple attack by florivores.

The ontogenetic stage in which plants are infested can influence the effects of attack on plant seed set (Rusman et al., 2020). In our field experiments, plants were exposed to attackers at the onset of flowering, and although the initial attack may have been minor for the plant, the attackers that settle on the flowering plant may multiply or develop to more voracious developmental stages while altering patterns of plant phenotypic responses. These attackers can potentially impact the composition of the inflorescence-associated community and seed production (Lucas-Barbosa et al., 2013; Pashalidou et al., 2013; Rusman et al., 2019b; Stam et al., 2018). When exposed to single or simultaneous dual attack by *B. brassicae* aphids, *P. brassicae* caterpillars and Xcr bacteria, which mostly attacked inflorescences, *B. nigra* plants produced similar numbers of seeds as control plants, which indicates that the plants compensated for damage and possible interference with mutualistic associations. Plants maintained interactions with both carnivores and pollinators despite changes in plant traits when exposed to single and dual attack to the inflorescences. Our results suggest that the resilience to attack of *B. nigra* may be supported by the chemical diversity that supports various mutualistic interactions of the plant (Gols et al., 2015; Lucas-Barbosa et al., 2017). Complex VOC blends of flowering plants likely evolved under selection pressure of both pollinators and herbivores, and may limit pleiotropic effects (Schiestl, 2015; Schiestl et al., 2014). We can expect to observe such flexibility for plants that exploit interactions with diverse community members, and compensation in seed production upon folivory and florivory seems to be common in the Brassicaceae (Lucas-Barbosa, 2016). Moreover, exploring the physiological and ecological plasticity of plants will bring complementary insights on the strategies developed by plants to cope with insect and pathogen attack, and on the evolution of plant chemical traits.

## Supplementary Information

ESM 1 (DOCX 783 kb)

## References

[CR1] Adams RP (1995). Identification of essential oil components by gas chromatography / mass spectrometry.

[CR2] Bahana J, Karuhize G (1986). The role of *Diaeretiella rapae* (M'cintosh) (Hymenoptera: Braconidae) in the population control of the cabbage aphid, *Brevicoryne brassicae* L. (Hemiptera: Aphididae) in Kenya. International J Tropical Insect Sci.

[CR3] Blubaugh CK, Asplund JS, Eigenbrode SD, Morra MJ, Philips CR, Popova IE, Reganold JP, Snyder WE (2018). Dual-guild herbivory disrupts predator-prey interactions in the field. Ecology.

[CR4] Bruinsma M, Ijdema H, Van Loon JJA, Dicke M (2008). Differential effects of jasmonic acid treatment of *Brassica nigra* on the attraction of pollinators, parasitoids, and butterflies. Entomologia Experimentalis et Applicata.

[CR5] Bruinsma M, Lucas-Barbosa D, ten Broeke CJM, van Dam NM, van Beek TA, Dicke M, van Loon JJA (2014). Folivory affects composition of nectar, floral odor and modifies pollinator behavior. J Chem Ecol.

[CR6] Chrétien LTS, David A, Daikou E, Boland W, Gershenzon J, Giron D, Dicke M, Lucas-Barbosa D (2018). Caterpillars induce jasmonates in flowers and alter plant responses to a second attacker. New Phytol.

[CR7] Conner J, Neumeier R (1995). Effects of black mustard population size on the taxonomic composition of pollinators. Oecologia.

[CR8] Cusumano A, Weldegergis BT, Colazza S, Dicke M, Fatouros NE (2015). Attraction of egg-killing parasitoids toward induced plant volatiles in a multi-herbivore context. Oecologia.

[CR9] da Silva SEB, França JF, Pareja M (2016). Olfactory response of four aphidophagous insects to aphid- and caterpillar-induced plant volatiles. Arthropod Plant Interact.

[CR10] Dannon EA, Tamò M, Van Huis A, Dicke M (2010). Effects of volatiles from *Maruca vitrata* larvae and caterpillar-infested flowers of their host plant *Vigna unguiculata* on the foraging behavior of the parasitoid *Apanteles taragamae*. J Chem Ecol.

[CR11] Desurmont GA, Laplanche D, Schiestl FP, Turlings TCJ (2015). Floral volatiles interfere with plant attraction of parasitoids: ontogeny-dependent infochemical dynamics in *Brassica rapa*. BMC Ecol.

[CR12] Dicke M, Simpson SJ, Mordue AJ, Hardie J (1999). Are herbivore-induced plant volatiles reliable indicators of herbivore identity to foraging carnivorous arthropods?. Proceedings of the 10th International Symposium on Insect-Plant Relationships.

[CR13] Dicke M, Baldwin IT (2010). The evolutionary context for herbivore-induced plant volatiles: beyond the ‘cry for help’. Trends Plant Sci.

[CR14] Dicke M, van Loon JJA, Soler R (2009). Chemical complexity of volatiles from plants induced by multiple attack. Nature Chem Biol.

[CR15] Dudareva N, Negre F, Nagegowda DA, Orlova I (2006). Plant volatiles: recent advances and future perspectives. Critical Rev Plant Sci.

[CR16] Erb M, Foresti N, Turlings TCJ (2010). A tritrophic signal that attracts parasitoids to host-damaged plants withstands disruption by non-host herbivores. BMC Plant Biol.

[CR17] Feltwell J (1982). Large white butterfly - the biology, biochemistry and physiology of *Pieris brassicae* (Linnaeus).

[CR18] Fritzsche-Hoballah ME, Turlings TCJ (2001). Experimental evidence that plants under caterpillar attack may benefit from attracting parasitoids. Evol Ecol Res.

[CR19] Gols R, Wagenaar R, Poelman EH, Kruidhof M, van Loon JJA, Harvey JA (2015). Fitness consequences of indirect plant defence in the annual weed, *Sinapis arvensis*. Funct Ecol.

[CR20] Hafez M (1961). Seasonal fluctuations of population density of the cabbage aphid, *Brevicoryne brassicae* (L.), in the Netherlands, and the role of its parasite, *Aphidius* (*Diaeretiella*) *rapae* (Curtis). Tijdschrift Over Plantenziekten.

[CR21] Hilker M, Meiners T, NJS L.M (2002). Induction of plant responses to oviposition and feeding by herbivorous arthropods: a comparison. Proceedungs of the 11th international symposium on insect-plant relationships.

[CR22] Irwin RE, Adler LS (2006). Correlations among traits associated with herbivore resistance and pollination: implications for pollination and nectar robbing in a distylous plant. Amer J Botany.

[CR23] Junker RR, Blüthgen N, Brehm T, Binkenstein J, Paulus J, Martin Schaefer H, Stang M (2013). Specialization on traits as basis for the niche-breadth of flower visitors and as structuring mechanism of ecological networks. Funct Ecol.

[CR24] Junker RR, Höcherl N, Blüthgen N (2010). Responses to olfactory signals reflect network structure of flower-visitor interactions. J Animal Ecol.

[CR25] Karowe DN, Schoonhoven LM (1992). Interactions among three trophic levels: the influence of host plant on performance of *Pieris brassicae* and its parasitoid, *Cotesia glomerata*. Entomol Exp et Appl.

[CR26] Kessler A, Baldwin IT (2001). Defensive function of herbivore-induced plant volatile emissions in nature. Science.

[CR27] Kessler A, Baldwin IT (2002). Plant responses to insect herbivory: the emerging molecular analysis. Annu Rev Plant Biol.

[CR28] Kessler A, Halitschke R (2007). Specificity and complexity: the impact of herbivore-induced plant responses on arthropod community structure. Curr Opinion Plant Biol.

[CR29] Kessler A, Halitschke R (2009). Testing the potential for conflicting selection on floral chemical traits by pollinators and herbivores: predictions and case study. Funct Ecol.

[CR30] Kessler A, Halitschke R, Poveda K (2011). Herbivory-mediated pollinator limitation: negative impacts of induced volatiles on plant-pollinator interactions. Ecology.

[CR31] Knauer AC, Bakhtiari M, Schiestl FP (2018). Crab spiders impact floral-signal evolution indirectly through removal of florivores. Nat Commun.

[CR32] Kroes A, van Loon JJA, Dicke M (2015) Density-dependent interference of aphids with caterpillar-induced defenses in *Arabidopsis*: involvement of phytohormones and transcription factors. Plant Cell Physiol 56:98–106. 10.1093/pcp/pcu150 10.1093/pcp/pcu150 25339349

[CR33] Krupnick GA, Weis AE, Campbell DR (1999). The consequences of floral herbivory for pollinator service to *Isomeris arborea*. Ecology.

[CR34] Lehtila K, Strauss SY (1997). Leaf damage by herbivores affects attractiveness to pollinators in wild radish, *Raphanus raphanistrum*. Oecologia.

[CR35] Li R, Schuman MC, Wang Y, Llorca LC, Bing J, Bennion A, Halitschke R, Baldwin IT (2018). Jasmonate signaling makes flowers attractive to pollinators and repellant to florivores in nature. Journal of Integrative Plant Biology.

[CR36] Li R, Wang M, Wang Y, Schuman MC, Weinhold A, Schäfer M, Jiménez-Alemán GH, Barthel A, Baldwin IT (2017). Flower-specific jasmonate signaling regulates constitutive floral defenses in wild tobacco. Proc Natl Acad Sci.

[CR37] Li Y, Weldegergis BT, Chamontri S, Dicke M, Gols R (2017). Does aphid infestation interfere with indirect plant defense against Lepidopteran caterpillars in wild cabbage?. J Chem Ecol.

[CR38] Lucas-Barbosa D (2016). Integrating studies on plant-pollinator and plant-herbivore interactions. Trends Plant Sci.

[CR39] Lucas-Barbosa D, Dicke M, Kranenburg T, Aartsma Y, van Beek TA, Huigens ME, van Loon JJA (2017). Endure and call for help: strategies of black mustard plants to deal with a specialized caterpillar. Funct Ecol.

[CR40] Lucas-Barbosa D, Poelman E, Aartsma Y, Snoeren TL, van Loon JJA, Dicke M (2014) Caught between parasitoids and predators – survival of a specialist herbivore on leaves and flowers of mustard plants. J Chem Ecol 40:621–631. 10.1007/s10886-014-0454-9 10.1007/s10886-014-0454-9 24888744

[CR41] Lucas-Barbosa D, van Loon JJA, Dicke M (2011). The effects of herbivore-induced plant volatiles on interactions between plants and flower-visiting insects. Phytochem.

[CR42] Lucas-Barbosa D, van Loon JJA, Gols R, van Beek TA, Dicke M (2013). Reproductive escape: annual plant responds to butterfly eggs by accelerating seed production. Funct Ecol.

[CR43] Machmud M (1982) *Xanthomonas campestris* pv. *amoraciae* the causal agent of *Xanthomonas* leaf spot of Crucifers (Cabbage, Louisiana), Vol. PhD Thesis: Louisiana State University and Agricultural & Mechanical College, Louisiana

[CR44] Mattiacci L, Dicke M (1995). Host-age discrimination during host location by *Cotesia glomerata*, a larval parasitoid of *Pieris brassicae*. Entomol Exper Appl.

[CR45] McCall AC, Irwin RE (2006). Florivory: the intersection of pollination and herbivory. Ecol Lett.

[CR46] McCulloch L (1929). A bacterial leaf spot of horse-radish caused by bacterium *campestre* var. *armoraciae*, N. var. J Agric Res.

[CR47] Muhlemann JK, Klempien A, Dudareva N (2014). Floral volatiles: from biosynthesis to function. Plant Cell Environ.

[CR48] Pareja M, Qvarfordt E, Webster B, Mayon P, Pickett J, Birkett M, Glinwood R (2012). Herbivory by a phloem-feeding insect inhibits floral volatile production. PLoS One.

[CR49] Pashalidou FG, Lucas-Barbosa D, van Loon JJA, Dicke M, Fatouros NE (2013). Phenotypic plasticity of plant response to herbivore eggs: effects on resistance to caterpillars and plant development. Ecology.

[CR50] Ponzio C (2016) Plants under dual attack : consequences for plant chemistry and parasitoid behavior, Vol. PhD thesis: Laboratory of Entomology (ed. Waheningen University, Wageningen

[CR51] Ponzio C, Gols R, Pieterse CMJ, Dicke M (2013). Ecological and phytohormonal aspects of plant volatile emission in response to single and dual infestations with herbivores and phytopathogens. Funct Ecol.

[CR52] Ponzio C, Gols R, Weldegergis BT, Dicke M (2014). Caterpillar-induced plant volatiles remain a reliable signal for foraging wasps during dual attack with a plant pathogen or non-host insect herbivore. Plant Cell Environ.

[CR53] Ponzio C, Weldegergis BT, Dicke M, Gols R (2016). Compatible and incompatible pathogen-plant interactions differentially affect plant volatile emissions and the attraction of parasitoid wasps. Funct Ecol.

[CR54] Rostás M, Ton J, Mauch-Mani B, Turlings TCJ (2006). Fungal infection reduces herbivore-induced plant volatiles of maize but does not affect naïve parasitoids. J Chem Ecol.

[CR55] Rusman Q, Karssemeijer PN, Lucas-Barbosa D, Poelman EH (2019). Settling on leaves or flowers: herbivore feeding site determines the outcome of indirect interactions between herbivores and pollinators. Oecologia.

[CR56] Rusman Q, Lucas-Barbosa D, Hassan K, Poelman EH (2020). Plant ontogeny determines strength and associated plant fitness consequences of plant-mediated interactions between herbivores and flower visitors. J Ecol.

[CR57] Rusman Q, Lucas-Barbosa D, Poelman EH (2018). Dealing with mutualists and antagonists: specificity of plant-mediated interactions between herbivores and flower visitors, and consequences for plant fitness. Functional Ecol.

[CR58] Rusman Q, Poelman EH, Nowrin F, Polder G, Lucas-Barbosa D (2019). Floral plasticity: herbivore-species-specific induced changes in flower traits with contrasting effects on pollinator visitation. Plant Cell Environ.

[CR59] Schiestl FP (2010). The evolution of floral scent and insect chemical communication. Ecol Lett.

[CR60] Schiestl FP (2015). Ecology and evolution of floral volatile-mediated information transfer in plants. New Phytol.

[CR61] Schiestl FP, Johnson SD (2013). Pollinator-mediated evolution of floral signals. Trends Ecol Evol.

[CR62] Schiestl FP, Kirk H, Bigler L, Cozzolino S, Desurmont GA (2014). Herbivory and floral signaling: phenotypic plasticity and tradeoffs between reproduction and indirect defense. New Phytol.

[CR63] Schuman MC, Barthel K, Baldwin IT (2012). Herbivory-induced volatiles function as defenses increasing fitness of the native plant *Nicotiana attenuata* in nature. Elife.

[CR64] Stam JM, Dicke M, Poelman EH (2018). Order of herbivore arrival on wild cabbage populations influences subsequent arthropod community development. Oikos.

[CR65] Stam JM, Kroes A, Li Y, Gols R, van Loon JJA, Poelman EH, Dicke M (2014) Plant interactions with multiple insect herbivores: from community to genes. Annu Rev Plant Biol 65:689–713. 10.1146/annurev-arplant-050213-035937 10.1146/annurev-arplant-050213-035937 24313843

[CR66] Stitz M, Hartl M, Baldwin IT, Gaquerel E (2014). Jasmonoyl-l-isoleucine coordinates metabolic networks required for anthesis and floral attractant emission in wild tobacco (*Nicotiana attenuata*). Plant Cell.

[CR67] Triba MN, Le Moyec L, Amathieu R, Goossens C, Bouchemal N, Nahon P, Rutledge DN, Savarin P (2015). PLS/OPLS models in metabolomics: the impact of permutation of dataset rows on the K-fold cross-validation quality parameters. Mol BioSyst.

[CR68] Turlings TCJ, Erb M (2018). Tritrophic interactions mediated by herbivore-induced plant volatiles: mechanisms, ecological relevance, and application potential. Annu Rev Entomol.

[CR69] van Loon JJA, de Boer JG, Dicke M (2000). Parasitoid-plant mutualism: parasitoid attack of herbivore increases plant reproduction. Entomol Exper Appl.

[CR70] Vaughn TT, Antolin MF, Bjostad LB (1996). Behavioral and physiological responses of *Diaeretiella rapae* to semiochemicals. Entomol Exper Appl.

[CR71] Vicente JG, Everett B, Roberts SJ (2006). Identification of isolates that cause a leaf spot disease of *Brassica* as *Xanthomonas campestris* pv. *raphani* and pathogenic and genetic comparison with related pathovars. Phytopathology.

[CR72] Wiskerke JSC, Vet LEM (1994). Foraging for solitarily and gregariously feeding caterpillars: a comparison of two related parasitoid species (Hymenoptera: Braconidae). J Insect Behav.

[CR73] Wright GA, Schiestl FP (2009). The evolution of floral scent: the influence of olfactory learning by insect pollinators on the honest signalling of floral rewards. Funct Ecol.

[CR74] Zangerl AR, Berenbaum MR (2009). Effects of florivory on floral volatile emissions and pollination success in the wild parsnip. Arthropod Plant Interact.

[CR75] Zhang PJ, Zheng SJ, van Loon JJA, Boland W, David A, Mumm R, Dicke M (2009). Whiteflies interfere with indirect plant defense against spider mites in *Lima bean*. Proc Natl Acad Sci U S A.

