# Supplementary material for: Multiple Attack to Inflorescences of an Annual Plant Does Not Interfere with the Attraction of Parasitoids and Pollinators
Source: J Chem Ecol. 2021 Jan 28;47(2):175–91. doi: 10.1007/s10886-020-01239-6 (PMC7904547; doi:10.1007/s10886-020-01239-6)
Supplement: Supplementary file 1 — (DOCX 783 kb) [file 10886_2020_1239_MOESM1_ESM.docx]

ELECTRONIC SUPPLEMENTARY MATERIAL

Journal of Chemical ecology

MULTIPLE ATTACK TO INFLORESCENCES OF AN ANNUAL PLANT DOES NOT INTERFERE WITH THE ATTRACTION OF PARASITOIDS AND POLLINATORS

LUCILLE T.S. CHRÉTIEN^1,2,3,4*^(ORCID: 0000-0002-4400-1086), HESSEL VAN DER HEIDE^1^, LIANA O. GREENBERG^1,5^, DAVID GIRON^2^(ORCID: 0000-0001-8356-0983), MARCEL DICKE^1^(ORCID: 0000-0001-8565-8896), AND DANI LUCAS-BARBOSA^1,6^(ORCID: 0000-0003-3440-1040)

*^1^Laboratory of Entomology, Wageningen University, Droevendaalsesteeg 1, Radix building, 6708PB Wageningen, The Netherlands*

*^2^Institut de Recherche sur la Biologie de l’Insecte (IRBI), UMR 7261, CNRS/Université François-Rabelais de Tours, Avenue Monge, Parc Grandmont, 37200 Tours, France*

*^3^Biology Department, École Normale Supérieure de Lyon (ENS L), 46 Allée d’Italie, 69007 Lyon, France*

*^4^ Current address: School of Biological and Marine Sciences, University of Plymouth, Drake Circus, Plymouth, PL4 8AA, United-Kingdom*

*^5^Environmental studies department, New College of Florida, 5800 Bay Shore Rd, Sarasota, FL 34243, Florida, USA*

*^6^Current address: Bio-communication & Ecology, ETH Zürich, Schmelzbergstrasse 9, 8092 Zürich, Switzerland*

**corresponding author:* [*lucille.nat@orange.fr*](mailto:lucille.nat@orange.fr)*, 0033 6 88 63 64 87*

**
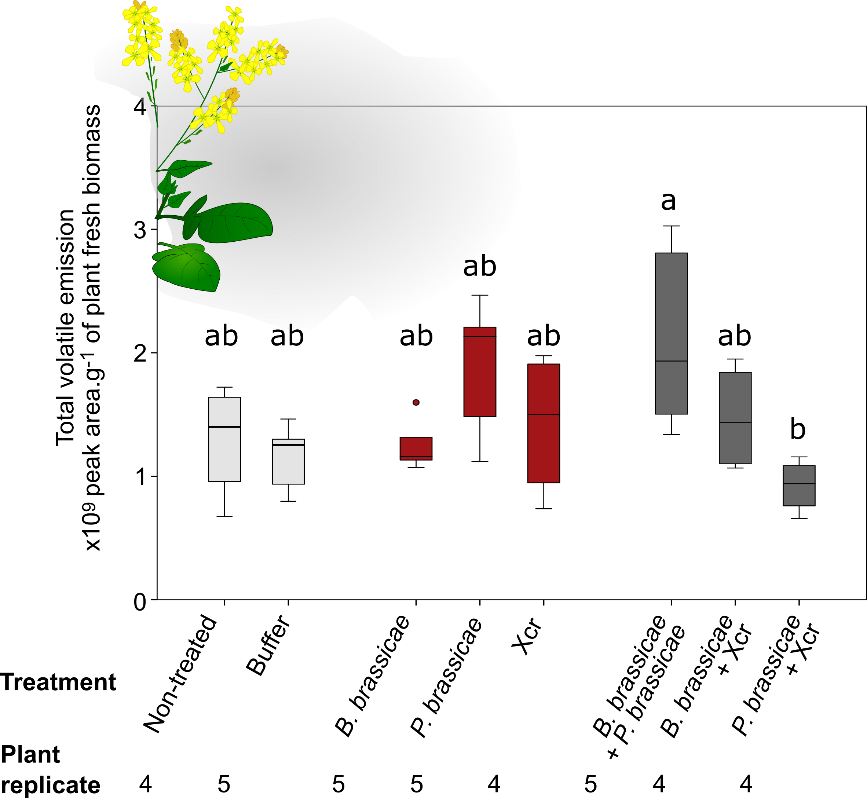
**

Fig. S1 Total volatile emission (x 10^9^ peak area g^-1^ of plant fresh biomass - median, interquartile range, full range) of aboveground parts of flowering *Brassica nigra* left untreated or exposed to buffer (light grey), to single (red) attack, and to dual attack (dark grey)

Volatile blends were collected for 1.5 h from aboveground parts of *B. nigra* exposed for 8 d to single or dual attack by aphids *Brevicoryne brassicae*, caterpillars *Pieris brassicae,* and/or *Xanthomonas campestris* pv. *raphani* (Xcr), exposed to buffer only or non-treated (controls). Xcr was inoculated in buffer, and all plants that were exposed to the insects received buffer too. Graph show the sum of the peak area of 59 volatiles that could be detected and quantified using chromatograms based on Total Ion Counts (TIC). Effect of treatments was analysed using a *Kruskal-Wallis* *test*: chi-square = 14.159, df = 7, P = 0.048), followed by a *Dunn-Bonferroni post-hoc* test to analyse differences between treatments. The significance level was set to α = 0.05. Outliers are represented by “ ˚ ” (further than 1.5 x Interquartile range).

**
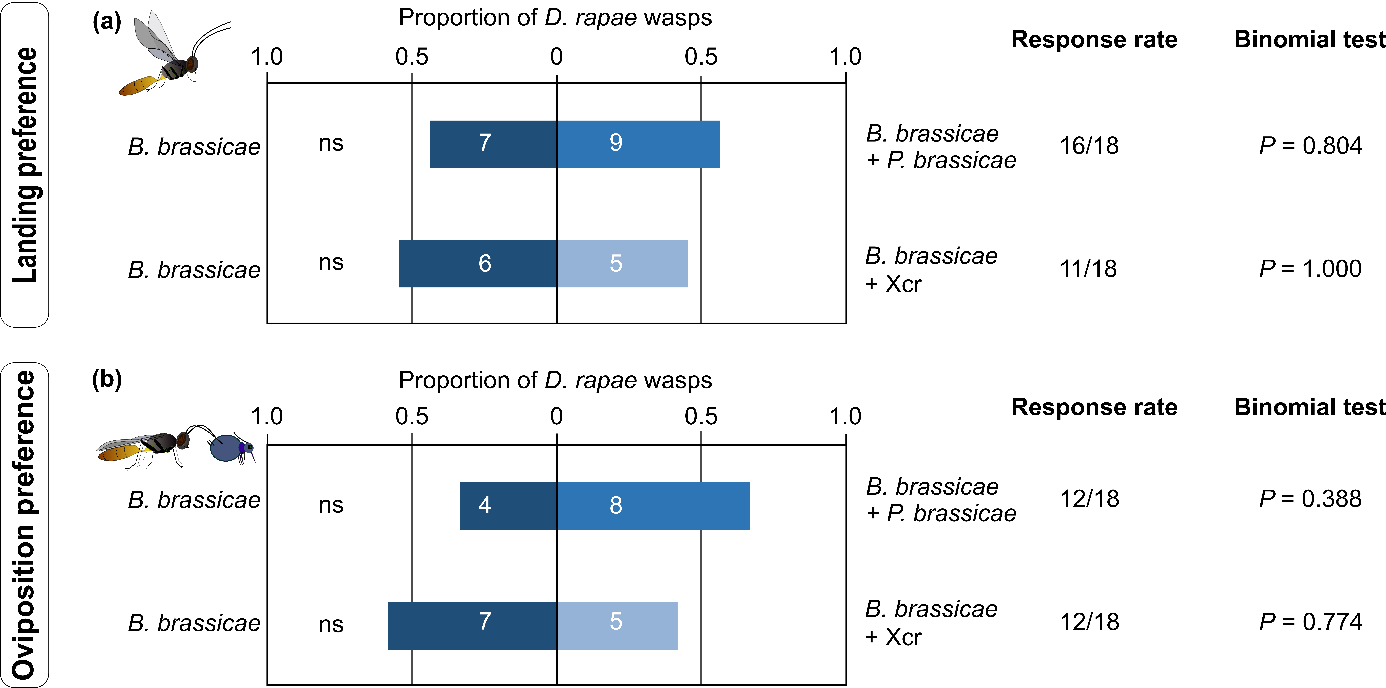
**

**Fig. S2 Proportion of *Diaeretiella rapae* parasitoids that landed and oviposited on flowering *Brassica nigra* plants exposed to single attack with aphids (host) *vs.* plants exposed to dual attack with aphids (host) plus caterpillars or plus bacteria (non-hosts).**

Preference of *D. rapae* wasps was tested in a two-choice assay in a greenhouse. Plants were either exposed to single attack by *Brevicoryne brassicae* aphids, or exposed to dual attack by *B. brassicae* plus *Pieris brassicae* caterpillars or by *B. brassicae* plus *Xanthomonas campestris* pv. *raphani* (Xcr) bacteria. Plants exposed to single and dual attack were combined two by two in a tent where a *D. rapae* wasp was released and left for 20h. We scored plants on which the wasps landed first (**a**) and plants that had highest count of parasitized aphids (mummies) (**b**). Response rate indicates the number of responding wasps over the number of tested wasps. Proportions were tested using a binomial test, and the significance level was set to α = 0.05.

**
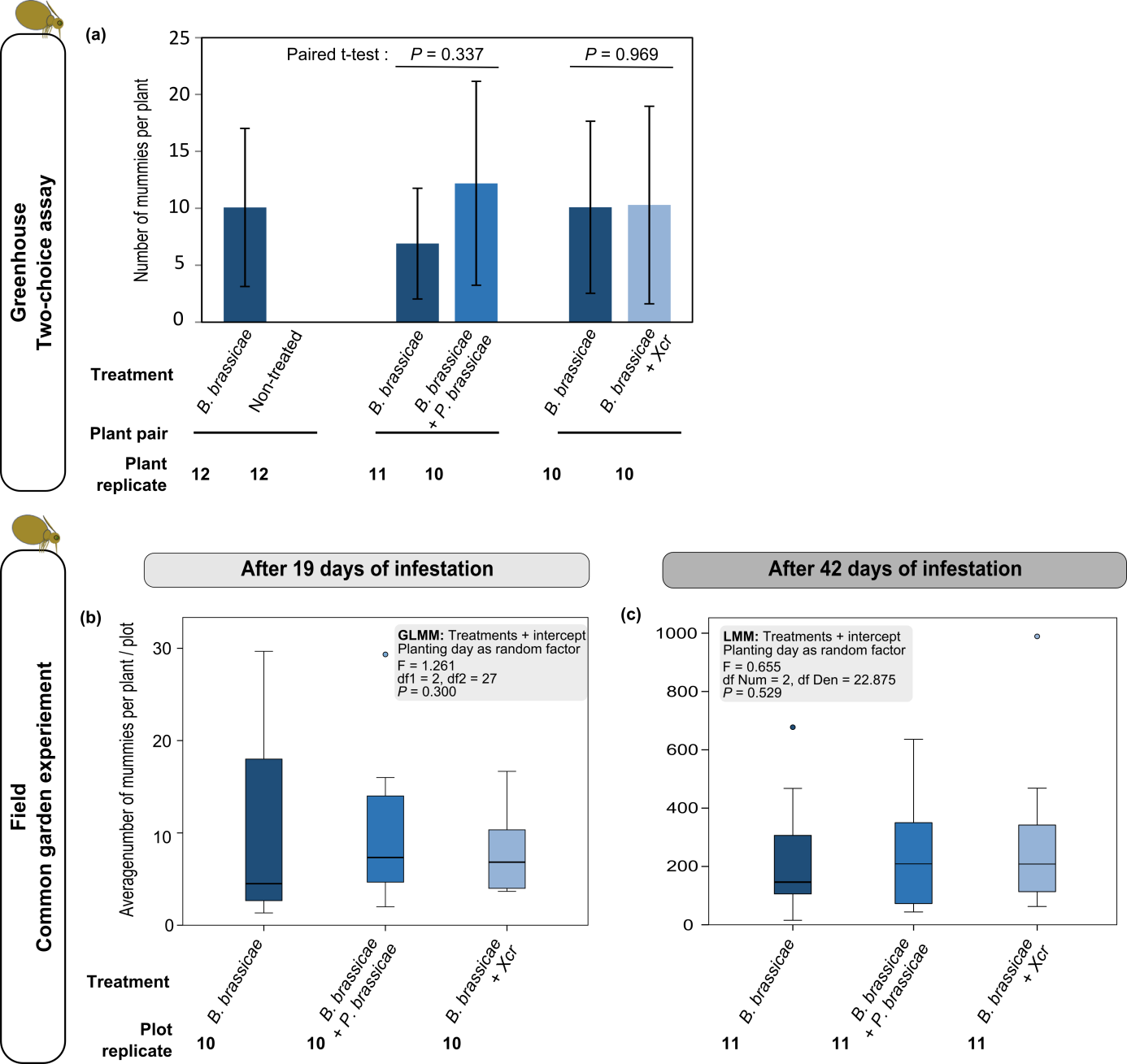
**

**Fig. S3 Number of aphids *Brevicoryne brassicae* that were effectively parasitized (mummies) on *Brassica nigra* plants exposed to single attack by *B. brassicae* (dark blue) or to dual attack by *B. brassicae* plus *Pieris brassicae* (blue) or plus *Xanthomonas campestris* pv. *raphani* (Xcr) (light blue) in the greenhouse and in the field**

**(a)** Number of mummies (mean ± SD) in the greenhouse 2-choice assay. Plants infested with the host aphids only or with another non-host (*P. brassicae* caterpillars or Xcr bacteria) were exposed to *Diaretiella rapae* for 20 h. Mummies were counted after 7 ± 1 d. **(b, c)** Average number of mummies of aphids (mostly *B. brassicae*) recorded per plant within a plot (median, interquartile range, full range) in the common garden experiment (Wageningen, The Netherlands, 2015) after 19 d **(c)** and after 42 d of exposure to the attackers **(d)**. Plants were organized in plots of 5 plants, and the central plant of each plot was exposed to either single attack by *Brevicoryne brassicae* aphids, or to dual combinations of these aphids plus *P. brassicae* or plus Xcr. Mummies were counted on the central plants and two side plants per plot, and the number of mummies was averaged at the plot level. *D. rapae* is the main parasitoid of *B. brassicae* in The Netherlands. **(a, b, c)** Effect of exposure to attackers of the number of mummies was analysed with a *paired t-test*, a Linear Mixed Model (LMM), and a Generalized Linear Mixed Model (GLMM – based on a negative binomial distribution with Logit as link function). The significance level was set to α = 0.05. “Num” stands for numerators, “Den” stands for denominator. Outliers are represented by “ ˚ ” (further than 1.5 x Interquartile range).


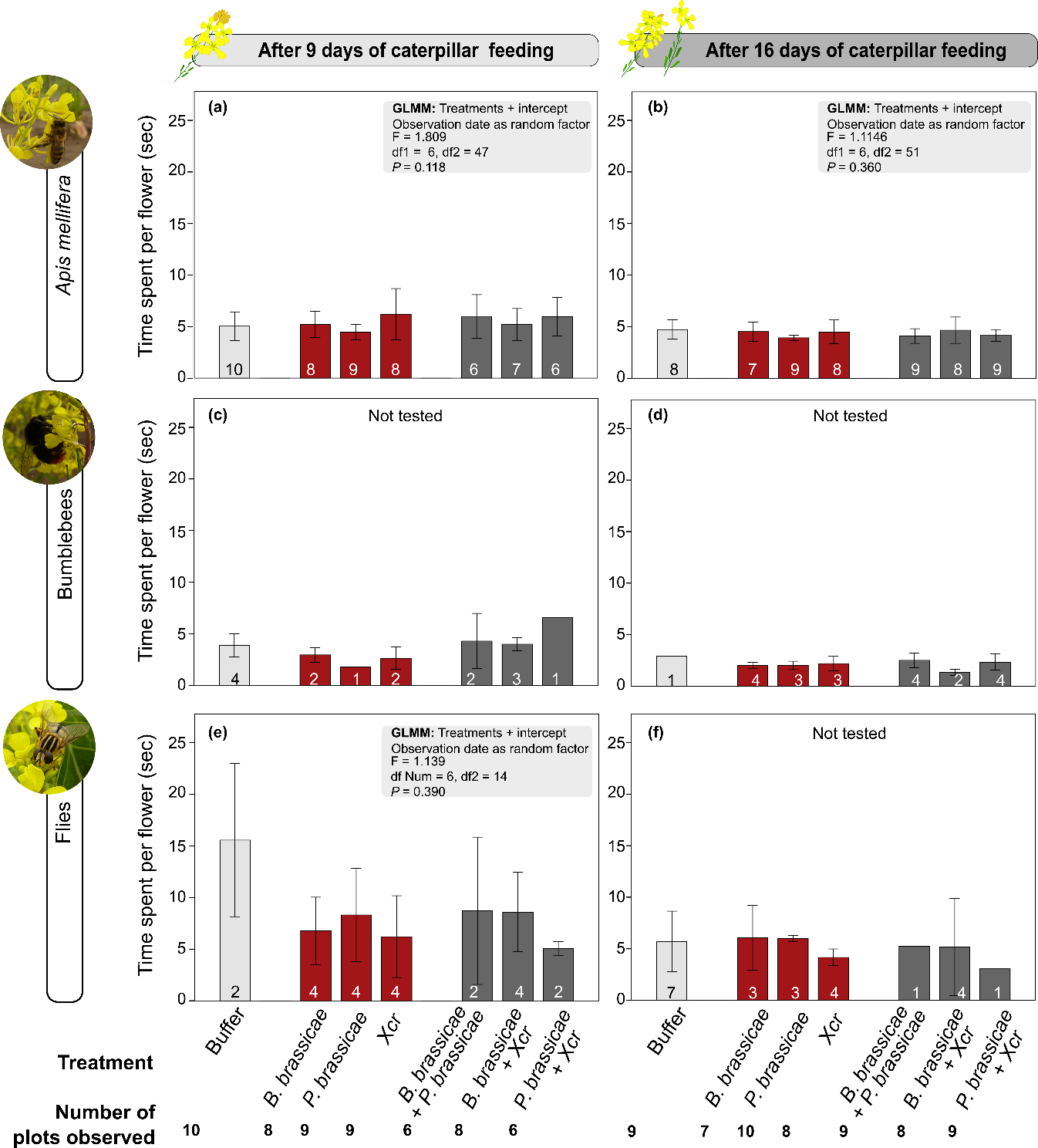


**Fig. S4 Time spent per flower (mean ± SD) by three types of pollinators on flowers of *Brassica nigra* plants exposed to buffer (light grey), single attack (red), and dual attack (dark grey)**

Pollinator visitation to *B. nigra* was recorded in a common garden experiment (Wageningen, The Netherlands, 2015). Plants were organized in plots of five plants, and the central plant of each plot was exposed to either single attack by *Brevicoryne brassicae* aphids, *Pieris brassicae* caterpillars or *Xanthomonas campestris* pv. *raphani* (Xcr), to dual combinations of those attackers, or to buffer only (control). We recorded the time that *Apis mellifera* (honeybee), bumblebees, and flies would spend per flower of a plot over 10 min of observation. Time spent per flower was recorded at two time points: 9 d and 16 d after *P. brassicae* started feeding from the plant (leaves); at both time points, caterpillars had started feeding from *B. nigra* flowers. Numbers in the bars indicate the number of plot replicate for each pollinator and time point. Effect of the treatments was analysed with a Generalized Linear Mixed Model (GLMM) based on a normal distribution, identity was set as link function. The significance level was set to α = 0.05. No statistical test was performed when there were fewer than two plot replicates for a treatment.

**Table S1** LIST OF VOLATILE COMPOUNDS COLLECTED FROM THE ABOVEGROUND PART OF FLOWERING *BRASSICA NIGRA* PLANTS EXPOSED FOR 8 DAYS TO SINGLE ATTACK (RED) WITH EITHER *BREVICORYNE BRASSICAE* APHIDS*, PIERIS BRASSICAE* CATERPILLARS OR *XANTHOMONAS CAMPESTRIS* PV. *RAPHANI* BACTERIA (XCR), TO DUAL ATTACK WITH TWO OF THESE ATTACKERS (DARK GREY), OR TO BUFFER (CONTROL – LIGHT GREY) AND OF NON-TREATED PLANTS (LIGHT GREY) IN THE GREENHOUSE, THE M/Z SELECTED TO MEASURE PEAK AREA WITH THE SINGLE ION COUNT METHOD (SIC), THE VIP VALUE OF THE COMPOUNDS IN PLS-DA ANALYSIS, AND PEAK AREA WITH THE SIC METHOD

**Table S2** TOTAL NUMBER OF POLLINATORS VISITING PLOTS OF *BRASSICA NIGRA* AFTER 9 D AND 16 D OF CATERPILLAR FEEDING IN THE COMMON GARDEN EXPERIMENT, WAGENINGEN, THE NETHERLANDS, 2015. WHEN COMPARING NUMBERS ACROSS TREATMENTS, NOTE THAT THE NUMBER OF REPLICATES N (NUMBER OF PLOTS) MIGHT BE DIFFERENT FROM ONE TREATMENT TO THE OTHER
